# Drug Inhibition Profile Prediction for NFκB Pathway in Multiple Myeloma

**DOI:** 10.1371/journal.pone.0014750

**Published:** 2011-03-07

**Authors:** Huiming Peng, Jianguo Wen, Hongwei Li, Jeff Chang, Xiaobo Zhou

**Affiliations:** 1 Department of Radiology, The Methodist Hospital Research Institute and Weill Cornell Medical College, Houston, Texas, United States of America; 2 School of Mathematics and Physics, China University of Geosciences, Wuhan, Hubei, P. R. China; 3 Department of Pathology, The Methodist Hospital Research Institute and Weill Cornell Medical College, Houston, Texas, United States of America; Memorial Sloan-Kettering Cancer Center, United States of America

## Abstract

Nuclear factor κB (NFκB) activation plays a crucial role in anti-apoptotic responses in response to the apoptotic signaling during tumor necrosis factor (TNFα) stimulation in Multiple Myeloma (MM). Although several drugs have been found effective for the treatment of MM by mainly inhibiting NFκB pathway, there are not any quantitative or qualitative results of comparison assessment on inhibition effect between different drugs either used alone or in combinations. Computational modeling is becoming increasingly indispensable for applied biological research mainly because it can provide strong quantitative predicting power. In this study, a novel computational pathway modeling approach is employed to comparably assess the inhibition effects of specific drugs used alone or in combinations on the NFκB pathway in MM and to predict the potential synergistic drug combinations.

## Introduction

Combined drug interventions are a common therapeutic strategy for complex diseases such as cancer [1]. As pointed out recently for cancer therapy [2], most therapies were initally developed as effective single agents and only later combined clinically. It is very important to previously predict the single drug-effect for effective drug selection related to specific diseases due to the huge number of drug agents. Moreover, a possible approach to the exploration of new theapeutic activities is not only present in individual drugs but also based on the exhaustive study of all possible combinations of compounds [3]. However, for drug combination strategy, time-consuming and expensive screening is needed to find promising combinatorial candidates from the vast number of natural and synthetic compounds available, and to see if they produce an appropriate biochemical or cellular effect [4]. Algorithms of making this drug combination screening faster, more effective and less expensive are in a continual development, such as synergistic combination screening [5], genetic algorithm [6] and floating forward selection [1]. However, all of these methods did not take insights into the drug effects on detailed signaling pathways. It is well-known that drug effects are governed by the intrinsic properties of the drug and the specific signal transduction network of the host such as disease cells. Predictability starts to become an important issue at the very begining of a discovery programme. Selection of a protein target is often based on evidence that the specific protein is significant in a pathway relevant to the disease of interest, this evidence perhaps being in the form of a knock-out showing an effect in changing cell physiology, and on evidence that the protein target's function can be affected by the binding of a drug molecule to it. This approach is deeply ingrained in the current intellectual furniture in drug discovery, and is characterised as the basis for ‘rational drug discovery’ [7]. Based on this concept, in this work we take TNFα-induced NFκB signaling pathway in MM as an example to show how to reach the aim of ‘rational drug discovery’ by combining computational pathway modeling approach with dynamic experimental data.

MM is the second most common hematologic malignancy, with about 15,000 new cases per year in USA, and remains incurable with a median survival of 3 to 5 years [8]. It is a plasma cell malignancy characterized by complex heterogeneous cytogenetic abnormalities. The bone marrow microenvironment promotes MM cell growth and resistance to conventional therapies [9]. Failure of myeloma cells to undergo apoptosis plays an important role in the accumulation of myeloma cells within the bone marrow. Several anti-apoptotic proteins and anti-apoptotic signaling cascades have been identified that contribute to the anti-apoptotic phenotype of the myeloma cells [8], [9], [10]. Actually, adhesion of myeloma cells to bone marrow stromal cells (BMSCs) triggers none-cytokine and cytokine-mediated tumour cell growth, survival, drug resistance and migration. MM cells binding to BMSCs upregulates cytokine secretion from both BMSCs and tumour cells. These cytokines activate major signaling pathways: extracellular signal-regulated kinase (ERK); Janus kinase 2 (JAK2)/signal transducer and activator of transcription 3 (STAT3); phosphatidylinositol 3-kinase (PI3K)/AKT; and NFκB. These pathways not only promote growth, survival and migration of MM cells, but also confer resistance to conventional chemotherapy. Targeting these mechanisms or inhibiting these pathways offers a potential therapeutic strategy to induce the apoptosis of MM cells and overcome drug resistance.

It has previously shown that canonical NFκB pathway in MM cells is mainly activated by TNFα [11], [12]. Several drugs effective for the treatment of MM, including bortezomib (BZM), thalidomide, lenalidomide and arsenic trioxide (ATO), have been found to block NFκB activation [13]. Therefore, blockade of TNFα-induced NFκB signaling by different single drugs or different drug combinations represent a novel therapeutic strategy in MM. However, at least to the best of our knowledge, there are no any quantitative or qualitative results of comparison assessment on inhibition effects between these different single drugs or drug combinations. So, we do not know how to choose drugs to inhibit the NFκB pathway, or we do not know which drug is the best one? What is the best dose for specific single drug? What is the best ratio and dose for specific drug combination? How about the inhibition effect if the drug combination is chosen with fixed ratio and dose? To answer these questions, a mass of biological experiments have to be designed to compare the inhition effects. However this tradional approach is time-consuming and expensive.

Computaional modeling is becoming increasingly indispensable for basic and applied biological research. Essentially, a mathematical model is a systematic representation of biological system, whose analysis can confer quantitative predicting power. One of the common applications of mathematical modeling is to analyze cellular networks systematically and another use of mathematical modeling has been demonstrated in devising strategies to control cellular dynamics. Therefore, the computational modeling is suitable for signaling pathway analysis and drug combination response analysis in our study.

**Figure 1:**
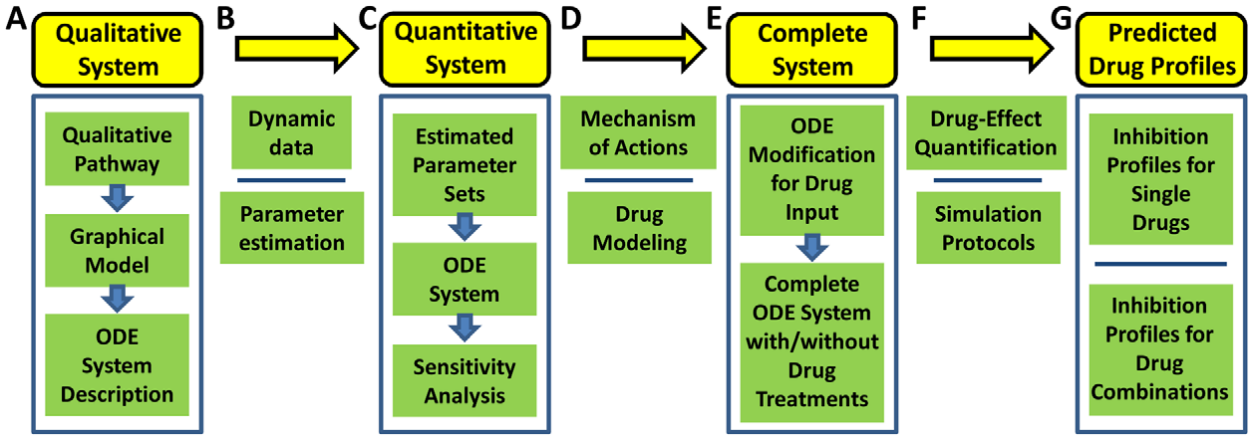
Workflow of the systematic procedure to predict drug-effects. (A) A qualitative system for general NFκB pathway is constructed based on the procedure from qualitative pathway, graphical model, to ODEs system description. (B) Dynamic experimental data are collected, and then optimization method is employed to estimate the unknown model parameters based on the dynamic data. (C) The quantitative system for specific NFκB pathway in MM is built after parameter estimation procedure, and then parameter sensitivity analysis is used to assess the stability of the constructed system. (D) The considered drugs are modeled into the quantitative system based on specific mechanism of actions. (E) The complete ODEs system with or without drug treatments is constructed after the ODEs modification for drug input. (F) Simulation protocols are designed to predict the drug effects based on the quantification methods. (G) Predicted drug profiles are presented for specific single drugs and drug combinations from model simulation.

In this paper, we try to employ the computational modeling approach to assess or predict the specific drug (used alone or in combination) responses on inhibition of NFκB pathway in MM. We firstly develop the computational model qualitatively, and then collect some specific experimental data to estimate the model parameters, and further design specific simulation protocols to predict the responses for single drugs and drug combinations. The workflow is presented in Figure 1. At first, a qualitative system for NFκB pathway is constructed based on the procedure beginning from qualitative pathway to graphical model and then to the ordinary differential equations (ODEs) system description. Then dynamic experimental data are collected, and optimization method is employed to estimate the unknown model parameters based on the dynamic experimental data. So, the quantitative system is built after the procedure of parameter estimation, and then parameter sensitivity analysis is used to asses the stability of the constructed system. After that, the considered drugs are modeled into the quantitative system based on specific mechanisms of actions and the complete ODEs system with or without drug treatments is constructed after the modification of ODEs with input of drugs. Then the simulation protocols are designed to predict the drug effects based on the quantification methods. Therefore, predicted drug profiles are presented for specific single drugs and drug combinations from model simulations, especially for the prediction of synergy on drug combinations based on Bliss combination index or Loewe isobologram quantification methods.

## Results

### Construction of qualitative system for general NFκB pathway

**Figure 2:**
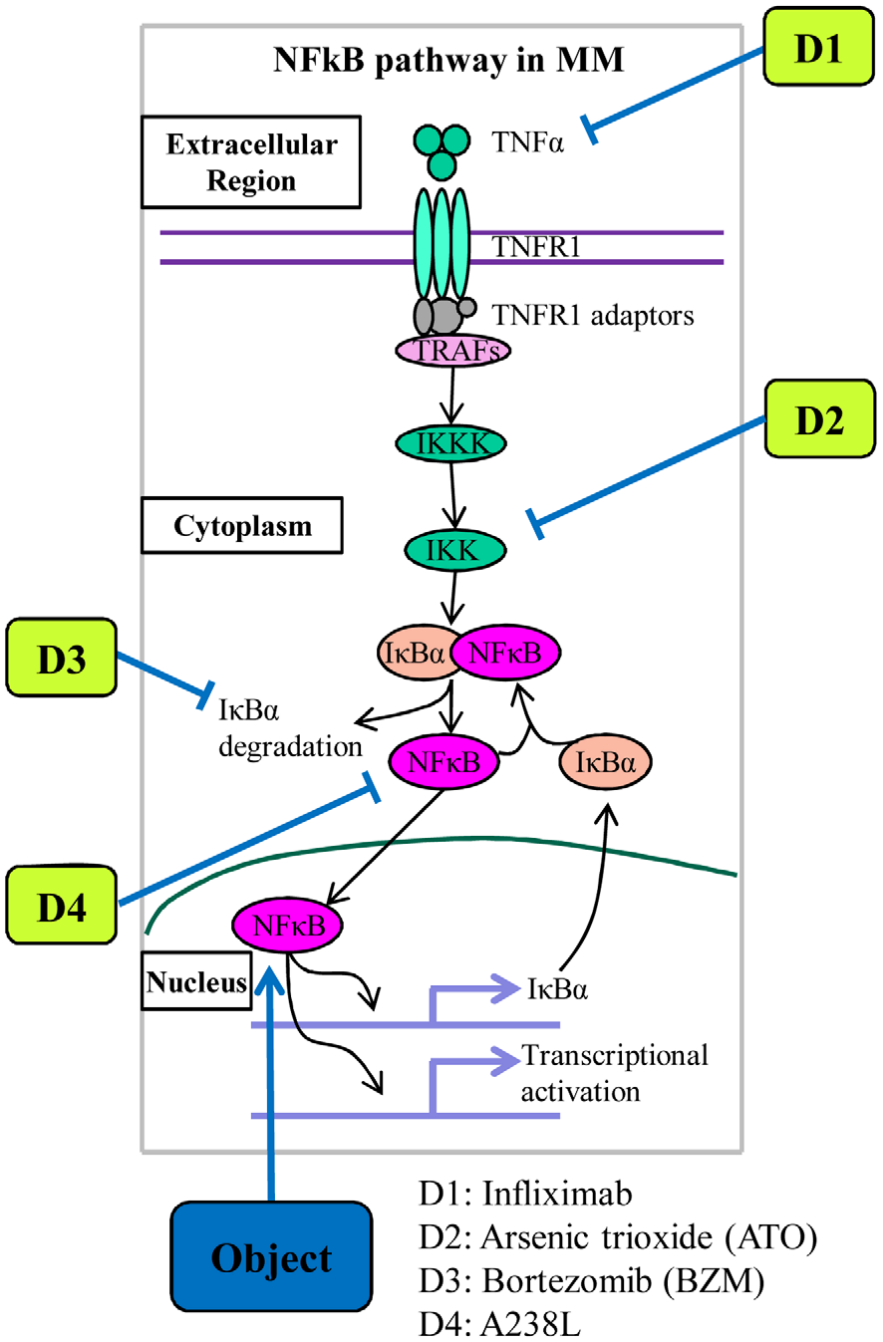
Qualitative NFκB pathway along with description of considered inhibitors. Firstly, the key cytokine TNFα binds to its receptor, leading to the recruitment of its adaptors and TRAFs, to form a complex which phosphorylates and activates IKKK, and the phosphorylated IKKK further activates IKK, leading to the phosphorylation and subsequent degradation of IκBα by 26 s proteasome. The direct consequence is the translocation of NFκB from the cytoplasm into the nucleus, leading to transcription of target genes. Meanwhile, NFκB also activates its own inhibitor, IκBα, giving rise to a negative feedback control [28]. By the way, four kinds of specific inhibitors with different targets are also described along with the qualitative NFκB pathway for the purpose of simulation protocols.

**Figure 3:**
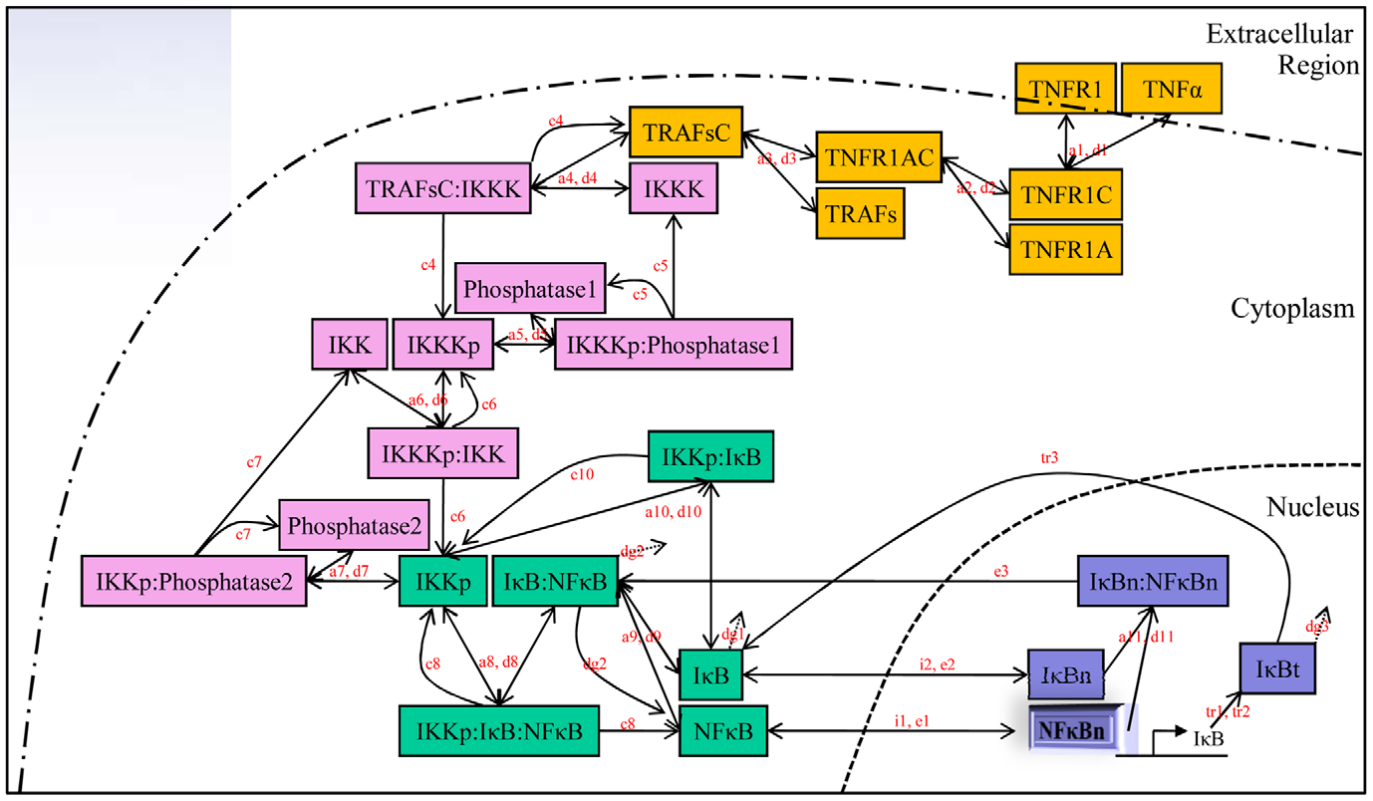
Graphical model for the reactions of NFκB pathway. Based on the different functions of components in the pathway, the whole system can be divided into four sub-systems. Different sub-systems in the pathway are shown in different colors. Yellow represents the TNFα receptor sub-system, pink represents the IKK phosphorylation cascade sub-system, green represents the cytoplasmic IKK-IκB-NFκB sub-system, and blue represents the nuclear IκB-NFκB sub-system, respectively. All of the model parameters are also shown at the side of the corresponding reaction arrows, and the symbols are chosen based on the different meanings, for example, symbol ‘a’ means association or binding rate, ‘d’ means dissociation rate, ‘c’ means catalysis rate, and so on.

To understand the interaction mechanisms of various molecular species in the NFκB activation module, we model this dynamical system using a set of ODEs, which can be used to systematically describe the time dynamics of concentrations for all the components in the pathway. For this purpose, the primary step is usually to construct the qualitative system. Firstly, the qualitative NFκB pathway collected from biological literatures is described (see Figure 2). Based on the qualitative pathway, the graphical model is then constructed (see Figure 3), and this model give us all of the details about the considered NFκB pathway including all of the reactions and all of the molecules related to the pathway and also all of the symbols of parameters in the ODEs model. In fact, this model provides us a clear idea on how to build the whole ODEs system for this model. Further, the detailed computational model with ODEs system is developed based on this graphical model (see Materials and Methods).

To facilitate the development of the computational model for NFκB pathway in MM, the following basic assumptions are made firstly.

- The cytoplasm can be considered as a uniform mixture in which all component molecules are uniformly distributed and they can access to each other with equal probability. And this assumption reduces the complexity of biochemical reaction modeling by considering only temporal changes of molecules rather than their localization.
- The law of mass action was used for presentation of all of the reactions in our model mainly including the binding-dissociation reactions and the enzymatic reactions. Although the commonly used reaction model for enzymatic reaction is the Michaelis-Menten equation which is the famous simplification of the law of mass action, we only use the classic law of mass action for all of the enzymatic reactions in the pathway modeling.
- In the pathway, IKKα and IKKβ were called the same name IKK and we did not explore their different functions no matter what in canonical or noncanonical NFκB activation pathway.
- We did not consider the effects of inhibitor proteins IκBβ and IκBε because, under constitutive activity of IKK, NFκB does not directly induce re-synthesis of these proteins. Therefore, their presence becomes negligible in the steady state [14].
- We did not consider the reactions of the binding and dissociation between NFκB and the complex of IκBα and IKK which were also mentioned in [15].
- We did not specify the components about NFκB heterodimer isoforms and we just simply considered the single NFκB isoform p50/p65 in our model similarly as considered in other literatures [14], [15], [16].

### Construction of quantitative system for specific NFκB pathway in MM

From the description of Figure 3, using the law of mass action, we can build the whole ODEs system for the considered NFκB model in MM. Generally, there are total 26 components in the model and 26 ODEs, and the total number of the parameters in the model is 39. It is worth noting that this ODEs model is motivated, but different, from various computational models for NFκB pathway in literatures [14], [15], [16]. By referring to these literatures, we collect the parameter values and initial concentrations of the components on the model. As expected that the simulation results from this ODEs model with these parameters and initial value sets for cytoplasmic IκB and nuclear NFκB presented an oscillation phenomenon as shown in Figure S1. The model consists of a series of ODEs describing the time evolution of concentrations of various molecules and molecular complexes of interest. The ODEs model involving four sub-systems are described in Materials and Methods.

A direct attempt to use the existed model parameters to describe our experimental data obtained from the human MM.1S cell line as described in Materials and Methods did not yield satisfactory result and the result is shown in Figure S2, which was not unexpected since different experimental models can yield different model parameters, and also the determination of the model parameters of signaling pathways is subject to uncertainty and non-identifiability of kinetic parameters of the enzymes involved in signaling as mentioned in [17]. We therefore carried out parameter fitting of the model to the dynamic experimental data described in Materials and Methods. The whole parameter estimation procedure in this study is referred to the method presented in [17] and the optimization procedure is implemented using DBsolve software with the version 7.48 [18], [19]. We use the following formula for parameter estimation.
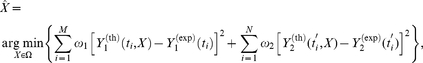
(1)where 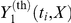 and 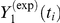 represent the theoretical and experimental data on the concentrations of IκB with time-points 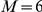, at 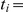
0, 5, 10, 15, 20 and 30 minutes; similarly, 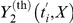 and 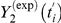 represent the theoretical and experimental data on the concentrations of nuclear NFκB with time-points 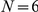, at 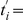
0, 10, 20, 30, 60 and 120 minutes. The weights 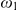 and 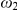 are used to scale the two square errors into the equal level, herein they are set as 
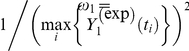
 and 
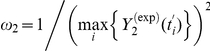
 respectively. 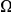 represents the candidate of parameter space for optimization procedure, in which the search space for each parameter is fixed between 0 and 1.

**Figure 4:**
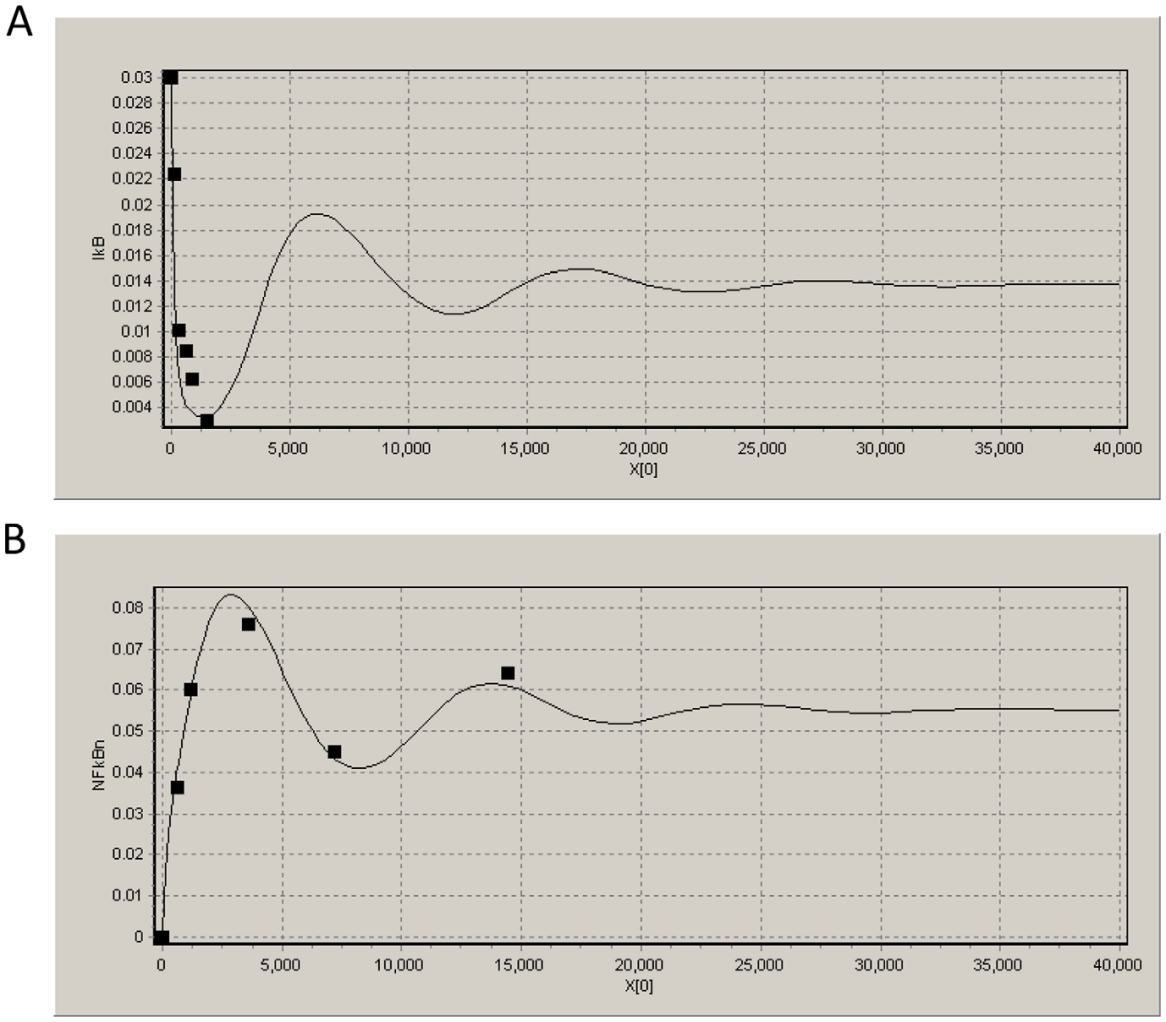
Data fitting results. This is the data fitting results for cytoplasmic IκB (A) and nuclear NFκB (B). Black box and solid curve represent the experimental data point and simulated results from the model after parameter estimation, respectively. In the coordinate system, X and Y axes present time and concentration, respectively.

In this procedure, the square error between the experimental and theoretical data is adopt for the cost function and then the Hook-Jeevse algorithm [20] is adopt to minimize the cost function in Equation (1). It is worth noting that all of the parameters for TNFα receptor and IKK phosphorylation cascade sub-systems and all of the initial concentration values in the pathway are kept the same as those in the literatures, and we use this procedure to fit the parameters to the experimental MM data for cytoplasmic IKK-IκB-NFκB sub-system and nuclear IκB-NFκB sub-system, because the reactions in these two sub-systems are specifically dependent on the type of cell line. Therefore, the total number of estimated parameters in this study is reduced to 21 from 39. In the procedure of optimization, the initial values of 21 estimated parameters are generated randomly between 0 and 1, and the desired square error is set at 0.01. In order to analyze the convergence of the optimization algorithm and to obtain the optimal estimation results, we execute the program for twenty times with different initial values. All of the results perform good convergence targeting the desired error, although the speed of convergence is not so fast with the average convergence time being about 7 hours. The final estimation results for the parameters are obtained by using the average of all the runs with the average square error being 0.0088. The fitting curves on the model can be seen from Figure 4 which shows the satisfied fitting results for the cytoplasmic IκB and nuclear NFκB concentration data after parameters estimation. The summary for all of the parameters is listed in Table S1, and Table S2 shows the summary for all of the initial concentrations in the model. Although there exist some differences on the model parameters between our fitted model and the model collected from literatures, the fitted model can reflect the experimental data well. Therefore, we will use this model for the further analysis in our study.

**Figure 5:**
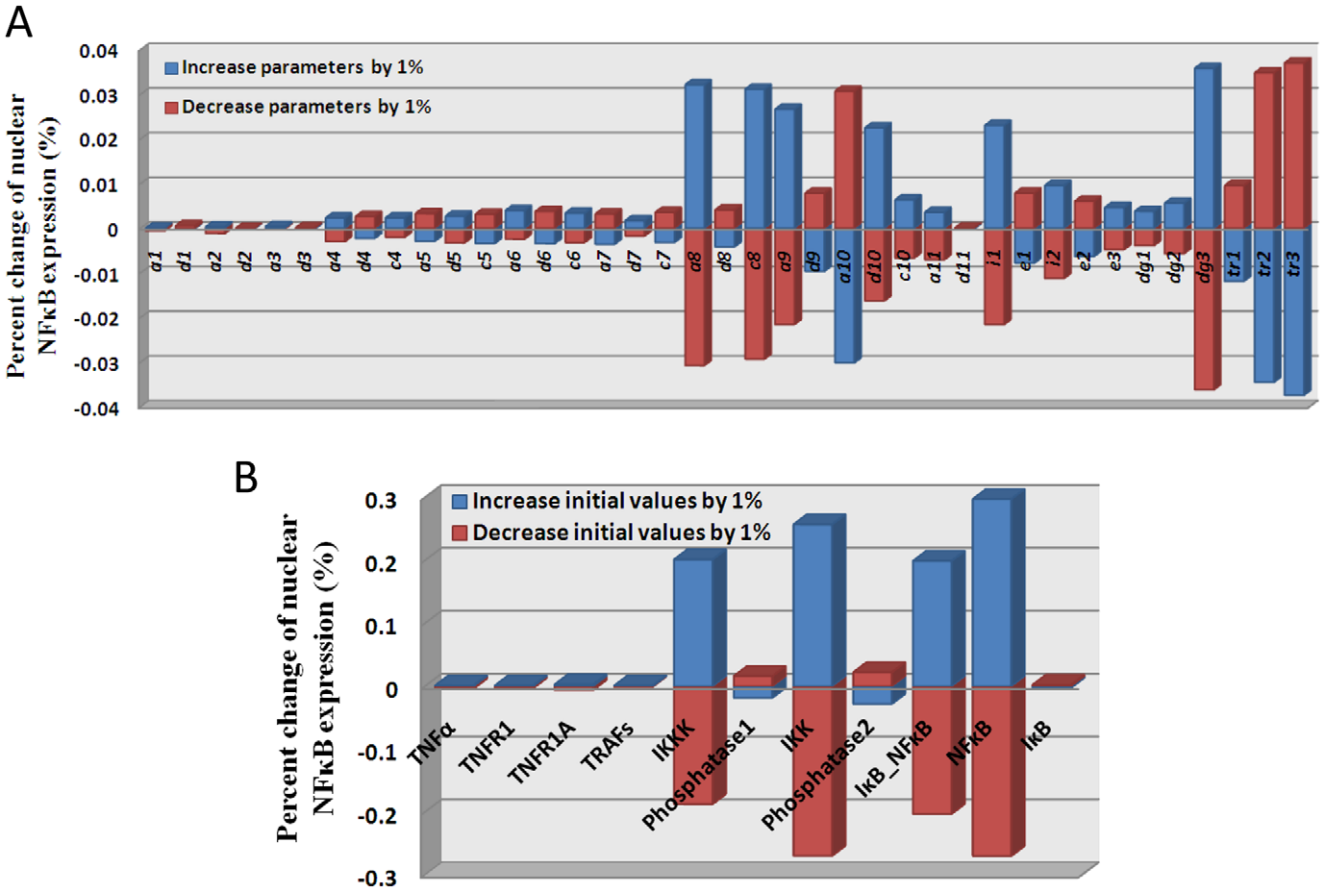
Parameter sensitivity analysis of the model. The above subfigure (A) shows the result of model sensitivity on total 39 kinetic parameters, and the below subfigure (B) shows the result of model sensitivity on 11 initial concentrations of corresponding molecules in the model. The results show the stability of the constructed pathway model and also give some suggestions on what are key kinetic parameters and molecules in the model.

Parameter sensitivity analysis is a tool to quantitatively determine the effect that specific parameters on the output. To understand the relationship between system responses and variations in individual model parameter values, local parameter sensitivity analysis was performed. The sensitivity coefficient (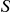) is defined as follows:
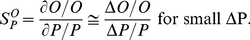
(2)Where 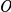 is the system output, i.e. the nuclear NFκB expression, and 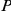 is the set of model parameters involving 39 kinetic parameters and 11 initial concentrations. Individual parameters were altered (i.e. increased or decreased) a little bit individually by 1% from their estimated values, and resulting changes in system output (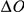) were determined. The resulting expression essentially denotes the percentage change in output resulting from 1% change in parameter 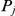. The results of sensitivity analysis on total 39 kinetic parameters and total 11 initial concentrations are shown in Figure 5. The results show that the model is more sensitive to a few parameters, i.e. 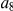, 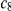, 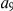, 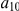, 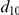, 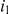, 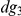, 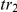 and 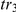, than the other parameters, and the results also show that the model is more sensitive to a few initial concentrations, i.e. IKKK, IKK, the complex IκB:NFκB, and cytoplasmic NFκB, than the other initial concentrations, which give us some suggestions on what are the key kinetic parameters and molecules in the system. Note that the percentage changes of nuclear NFκB expression in all cases are less than 0.04%, which shows the constructed pathway model is very stable, especially for TNFα receptor sub-system and IKK phosphorylation cascade sub-system corresponding to the parameter set from 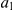 to 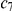 in Figure 5(A), which shows the rationality that all of the parameters in these two sub-systems are fixed before parameter estimation. All of the results for sensitivity analysis are shown in Figure 5.

### Development of a complete system for NFκB pathway in MM with or without drug treatments

Once we have built the quantitative mathematical model for NFκB pathway, different drugs with different targets should be modeled into the constructed ODEs system by specific mechanisms in order to study the different inhibition profiles on single drugs or drug combinations by simulating the model, meanwhile these protocols of simulation are also able to predict the optimal combination on the considered drugs. In this study, we just focus on the following four kinds of drugs, i.e. Infliximab, Aresenic tricide (ATO), Bortezomib (BZM) and A238L and we call them D1, D2, D3 and D4 for the purpose of simplification, and the corresponding targets are TNFα, IKKp, IκBα degradation and cytoplasm NFκB, respectively. Figure 2 provides the graphic idea for these inhibitors in NFκB pathway. The details for the mechanisms of actions and drug modeling procedure are presented in Materials and Methods.

### Inhibition percentage curves and single-drug evaluations

Once the considered drugs have been modeled into our ODEs system, we can simulate the whole model by changing the input of single drug dose, and then to predict the different steady output values for nuclear NFκB concentration corresponding to the input. By comparing these values with the control values (i.e. the nuclear NFκB concentrations in the case without drug input), the inhibition percentage curves on different single drugs can be calculated, meanwhile this kind of inhibition curve can be used as reference to assess the single drug effect. In detail, given the input of the specific single drug with dose 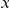, the corresponding inhibition percentage or inhibition rate 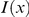 is defined as follows,
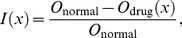
(3)where 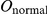 is the system output in the normal case, i.e. the nuclear NFκB expression in the case without drug input, which is fixed at 0.055 µM in this study according to the previously estimated model; 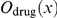 is the system output in the case with drug input, which can be obtained from the simulation of model.

**Figure 6:**
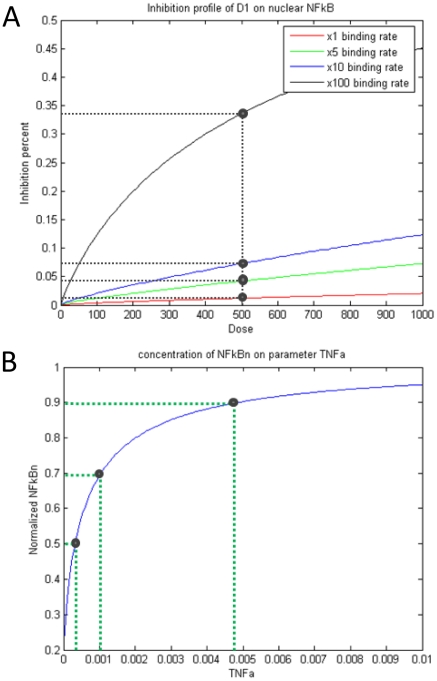
Nearly no effect for D1. (A) Several inhibition profiles of D1 on nuclear NFκB corresponding to different binding rates; (B) Normalized nuclear NFκB concentration curve on the initial concentration of TNFα. We change the drug dose in a huge range from 0 uM up to 1000 uM to look over the inhibition percentage. The red inhibition curve is based on the normal drug binding rate. It shows, throughout the dose range, the inhibition percentage is less than 3%, almost no effect. Then we magnify the binding rate by 5, 10 and 100 times, but the inhibition results are still not significant. So we claim it is nearly no effect for D1, it means that it is not a good idea using D1 to inhibit the NFkB pathway. Note that this result is consistent with the clinical result of very high expression of TNFα in MM.

It is shown from the simulation of single drug D1 with the normal binding rate that the inhibition effect is negligible regardless of the huge and unreasonable dose 1000 µM, as it can be seen from the bottom curve in Figure 6(A). It can be guessed spontaneously that this result may be due to the low rate of drug binding, so we magnify the binding rate by 5, 10 and 100 times higher than the normal one, then run the simulation again. The results in Figure 6(A) show that the inhibition effects are still very low and just about 2%, 4%, 8% and 34% corresponding to the different binding rates at fixed 500 µM dose. So, the influence of the binding rate is not significant to explain the ineffectiveness of D1. By another simulation, we try to seek the relationship between the nuclear NFκB concentration and the initial concentration of ligand TNFα. The predicted result shows that about 0.0003 µM, 0.001 µM and 0.0048 µM TNFα, i.e. about 0.15%, 0.5% and 2.4% of normal initial TNFα dose 0.2 µM, can sufficiently lead to 50%, 70% and 90% nuclear NFκB output comparing to the normal case, as it can be seen in Figure 6(B). This result suggests that the stimulus of TNFα with 0.2 µM concentration is largely redundant to stimulate the production of the nuclear NFκB, which is consistency with the clinical result of high expression of TNFα in MM. Therefore, we claim that D1 is nearly no effect to inhibit the NFκB pathway in MM due to the large redundancy of TNFα expression.

**Figure 7:**
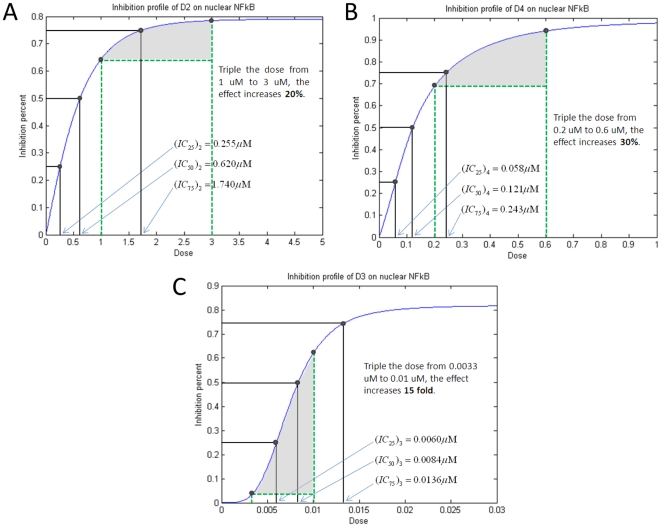
Different types of inhibition profiles on single drugs D2, D3 and D4. Different inhibition profiles on nuclear NFκB production by different single drugs D2 (A), D4 (B) and D3 (C). The above figures show two types of functions on the inhibition profiles, that is, hyperbolic type function for both D2 and D4, but sigmoidal type function for D3. It also shows that there exist extremely different characters between these two types of functions. For example, triple the D2 dose from 1 uM to 3 uM, the inhibition effect only increase 20%. Triple the D4 dose, it also only increase 30%. But, triple the D3 dose, it can produce 15 fold increase. By the way, from this profile, we can easily get the 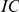 value prediction for different inhibition percentages, like 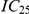, 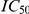, and 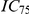. For example, 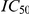 represents the concentration of a drug that is required for 50% inhibition. These 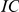 values will be used for the drug combination study.

It is shown from the inhibition profiles in Figure 7 that there exist different types of profiles for D2, D3 and D4. It can be concluded that D2 and D4 share the similar inhibition profile with hyperbolic type function, but D3 has the different inhibition profile with sigmoidal type function. Note that there exist some extremely different properties between these two types of functions, as pointed out in Figure 7 that tripling dose just increases the inhibition effect 20% and 30% for D2 and D4, but increases 15 fold of the effect for D3. From this character, to certain extent we can conclude that D3 is much better than D2 and D4 if we want to choose a single drug to inhibit the NFκB pathway. Of course, we omit some other factors, such as side-effect, economical consideration, and so on. It is worth noting that this drastic difference between these two types of inhibition profiles underscores the difficulty to predict by inspection what would be the “additive effect” when two drugs are combined at a given ratio. By the way, from this kind of profile, we can easily get the predicted 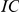 values for different inhibition percentages, like 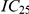, 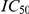 and 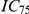, for example, 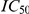 represents the concentration of a drug that is required for 50% inhibition. These 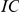 values will be used in the drug combination study.

### Combination index and drug combination evaluations

It is well-known that, for drug combination, two drugs working together maybe can produce an effect greater than the expected combined effect of the same agents used separately, and we call this case as synergy combination. Otherwise, we call the combination as additive effect (i.e. equivalent effect) or antagonism (i.e. less effect). In addition, different ratio combinations of dose for the same two drugs sometimes can produce totally different effects, such as one combination is synergistic but another is antagonistic. Therefore, it is also significant to predict the synergy combinations of dose ratios using computational model. Although a number of available mathematical combination indexes can be used to assess the effect of drug combination, in this study we prefer to select Bliss independence [21], because it is not only a famous synergy quantification method but also extremely convenient for calculation. Firstly, we briefly introduce the Bliss independence idea as follows. Let 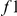, 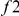 and 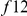 denote the effects for single drug 1, single drug 2 and the drugs 1&2 combination respectively, then it is firstly defined the combination as Bliss synergy if 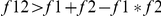, Bliss additive if 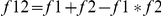, and Bliss antagonism if 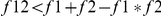. In this study, following the Bliss independence idea mentioned above, we then define a Bliss combination index as follows, 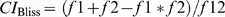. Given threshold_up and threshold_down, the effect of drug combination is defined as synergy if 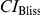 < threshold_down, and antagonism if 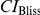 > threshold_up, otherwise additive. In this study, the thresholds are fixed as threshold_down = 0.99 and threshold_up = 1.01, i.e. 1% perturbation by noise is tolerated. In the simulation procedure, the Bliss combination index will be used to assess the synergy of drug combinations. In this study, the inhibition rate 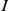 defined in Equation (3) will be applied as the index of the drug effect. So, we give the definition of the Bliss combination index in details here. For drug 1 and drug 2, given the system input with dose combination 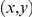, the corresponding Bliss combination index 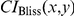 is defined as follows,
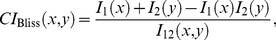
(4)where 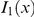 and 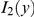 are the inhibition rates for the single drug 1 with dose 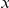 and the single drug 2 with dose 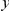, respectively, which are defined in Equation (3); 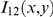 is the inhibition rate for the drug 1 & drug 2 combination with dose 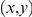, which has similar definition as mentioned in Equation (3).

**Figure 8:**
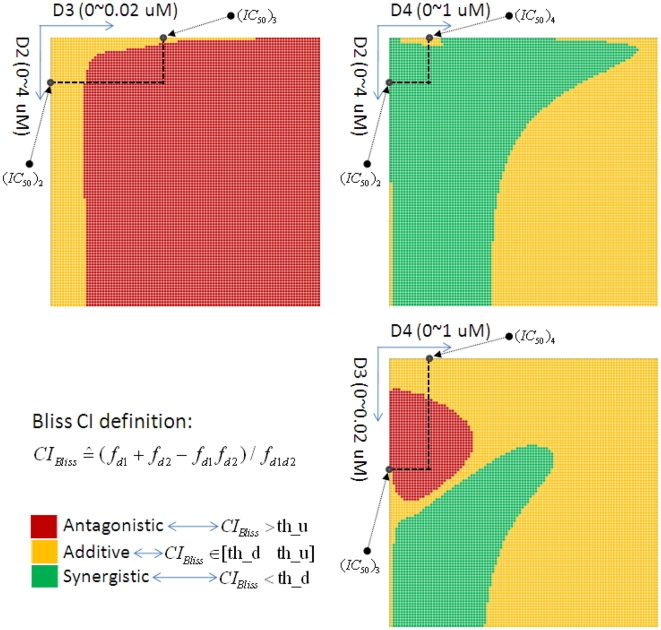
Synergy prediction on D2&D4 combination based on Bliss combination index. Heat maps of different drug combinations, i.e. D2&D3, D2&D4 and D3&D4, based on Bliss combination index to predict the synergistic region for combination. Different types of combination effects are shown in different color in the heat maps, and the description of definitions for Bliss combination index and three types of combination effects are also shown in the bottom-left.

Based on the prediction of inhibition profiles for D2, D3 and D4 shown in Figure 7, we choose suitable ranges of dose for each drug to analyze the drug combinations, i.e. 0∼4 µM for D2, 0∼0.02 µM for D3 and 0∼1 µM for D4. It is worth noting that the chosen dose ranges are consistent with biological consideration at least for D2 (ATO) and D3 (BZM). We evenly divide each range into 100 equal portions and then calculate the corresponding Bliss combination index defined previously for each combination. Note that the total number of dose combinations for each two-drug combination is equal to 10,000. The simulation results for heat-maps of Bliss combination index are shown in Figure 8. Note that the threshold parameters, i.e. threshold_up and threshold_down previously defined in the Bliss evaluation are fixed at 1.01 and 0.99 respectively, of course, other perturbations with more or less intensity are also considered for testing and the similar results also can be obtained. It can be found from Figure 8 that all of three drug combinations, i.e. D2&D3, D2&D4 and D3&D4, have different inhibition profiles corresponding to different dose combinations. For D2&D3, most of the dose combinations are detected as antagonistic effect because most regions display in red color in the corresponding heat map in Figure 8, and other small parts of combinations are detected as additive effect, and this result is also applicable if we just focus on the region within 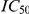 values. For D2&D4, synergistic effect is detected for most dose combinations fortunately, meanwhile no antagonistic effect has been detected and all the remains are additive. Moreover, almost all of the dose combinations within 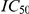 region are shown as synergistic. For D3&D4, all of three types of combination effects have been detected, but just additive and antagonistic effects are shown within 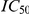 region. From these combination profiles, it can be concluded that the D2&D4 drug combination is the best choice, D2&D3 is the worst one and D3&D4 is the mediacy, meanwhile the predicted synergistic regions in D2&D4 and D3&D4 combinations are potentially helpful to conduct the clinical drug combination experiment.

## Discussion

As we mentioned in the previous text, inhibition of NFκB activation has been proposed as a potential therapeutic strategy in the treatment of MM. Although different drugs, such as the drugs considered in this work, with different targets can be used to inhibit the NFκB pathway, no detailed drug-effect profiles have been reported in literatures. So, the aim of this work is to comparably assess the inhibition profiles for specific single drugs and drug combinations, especially for the prediction of synergy on drug combinations. We used the computational pathway modeling combining with dynamic experiment data to do this work. At first, the dynamic experimental data are used to build the computational pathway system. Then the simulation protocols are figured out for this model simulation. For the study of single drug profile, we put single drug with adjustable dose one by one into the system, check the output, then compare it with the control case to get the profile. For the study of drug combination profile or synergy study, we put two drugs together with adjustable dose combination into the system, check the output, and then compare it with the control case to get the profile based on Bliss independence evaluation quantification method. Finally, the simulation results for the study of single drugs show that it is nearly no effect for D1 to inhibit the NFκB pathway, and it also show that there exist different types of functions for the inhibition profiles of single drugs D2, D3 and D4. The simulation results for drug combination study show that there exists strong synergy effect for D2&D4 combination, however strong antagonism effect has been predicted for D2&D3 combination. Note that the result for D2&D3 combination is consistent with our previous study in [22], [23] which suggested that although the synergy occurred on proliferation inhibition of human MM cells for D2&D3 drug combination treatment, this synergy effect was mainly reflected in JNK pathway rather than NFκB pathway. It is also worth noting that the D2&D4 combination has the potential to work in synergism by our model simulation although this predicted result has not been reported, and the validation by further biological experiment in our laboratory should take long time due to the procedure of cell culture. These predicted results can be used to instruct the experiment in biology.

In order to test the consistency of the predicted results for drug combinations, another synergy quantification method has also been employed for this purpose. As we know, the two most used reference models for quantifying synergy are Bliss independence [21] and Loewe additivity [24]. And, the Loewe additivity model, along with the associated graphical concept of the isobologram, is usually used by combining with Bliss independence to explore more information for the prediction of drug combinations. Herein, we briefly introduce the concept of Loewe synergy. The general visualized description of Loewe synergy can be seen from Figure S5, in which the combination index of Loewe synergy for drug 1 & drug 2 is defined as 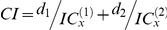, where 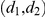 is the drug combination dose located in the combination contour line or isobologram, 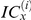 (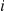 = 1, 2) denotes the x% percentage-based inhibition concentration of drug 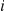 and 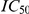 is the classic one as we mentioned previously. As mentioned in the sub-figure box of Figure S5, 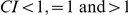 indicate Loewe synergism, additive effect, and antagonism, respectively. As it can be shown from Figure S5, as an example of Loewe synergy, that the red solid contour line is a 50% isobologram, i.e. the locus of 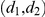 combination points producing the 50% inhibition, and we say that it has Loewe synergy for all the combination of drug 1 & drug 2 at all the combination ratios since the contour bows inward.

From the simulation of mathematical model, we calculate the Loewe isobolograms for different drug combinations based on different inhibition percentages. The results are presented in the Figure S6 for drug combinations D2&D3, D2&D4 and D3&D4 at inhibition concentrations 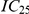, 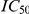 and 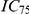. Using the concept of Loewe synergy, we can obtain some results from the Figure S6 that for drug combination D2&D3, only strong antagonism is presented because all the isobolograms 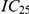, 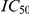 and 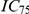 are outward strongly; for drug combination D2&D4, the weak antagonism is presented at 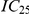 and 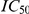, fortunately the strong synergism is presented in the case of 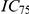 because the 75% isobologram is inward strongly; for drug combination D3&D4, the strong antagonism is presented at 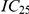 and 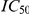, however all of three kinds of drug combination effects, i.e. synergism, additive effect and antagonism, are presented in the case of 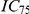, which means that it is able to produce different effects corresponding to different dose combinations. We conclude from the Loewe synergy analysis that both of the drug combinations D2&D4 and D3&D4 can produce synergy effect, but not for the combination D2&D3. This kind of result is consistent with that from Bliss independence quantification method, which may be potentially useful for the selection of drug combinations in the chemical therapy.

There exist two limitations in this current work. One is that only one key pathway (in this case, NFκB pathway induced by TNFα treatment) is considered here, and another is that the molecular output in the pathway (in this case, nuclear NFκB expression) is not linked to specific cell phenotypic behaviors in MM. At first, a pathway-centric approach remains incomplete because of the intricate cross talks among cell regulatory pathways [25]. Indeed, a given molecular component can be identified to be associated with or interact with multiple signaling. Pathways thus cannot properly be considered to operate in isolation of one another, as an alteration of one pathway can lead directly or indirectly to changes in others. To address this problem, a specific growing approach has been proposed in our laboratory used to expand the seed pathway (in this case, NFκB pathway) by combining protein-protein-interactions (PPI) information with Microarray data of MM cell line. In brief, given the set of interested genes and proteins as the seeds, we can construct the generic pathway map by growing those seeds based on the interaction database. Further, we will integrate the experimental data to determine the signaling process and positive/negative feedback loops in the expanded network. Finally, the single NFκB pathway can be expanded to multi-pathways in order to solve this problem. For the second limitation, most of the current work-like system modeling efforts aimed at predicting the effects of therapeutic perturbations of cell regulatory pathways, i.e. restricted its attention to predict molecular-level processes (in this case, nuclear NFκB expression). What is vital, of course, is to predict the effects of these perturbations on cell phenotypic functions at the very least. The most difficult problem is to connect the molecular-level pathway activities to the cell-level functional behaviors, even in absence of therapeutic perturbations. Fortunately, relational modeling methods, such as partial least squares regression [26] and quasi-non-parametric/generalized model [27], which both link the key phosphorylated proteins to the cell fate decisions using specific linear/non-linear functions, can be employed as the most effective approaches to solve this problem.

## Materials and Methods

### Dynamic experimental data

Although there were a few computational models for the NFκB pathway and most of the model parameters have been identified [14], [15], [16], all of these models did not focus on the specific MM cell line. In this study, we focus on the specific NFκB pathway in MM. So it is necessary to validate and rectify the model obtained from the literatures based on the experimental data produced from the specific human MM cell line. For this purpose, we have collected different types of data from literatures [11], [12] and also from our laboratory. We firstly point out that all of these data are produced from human MM.1S cell line stimulated with 0.2 uM TNFα, which is consistent with our model in this study because our model is also focused on MM with 0.2 uM TNFα stimulation. Herein we obtained some time-course experimental data on protein expression for key components of NFκB pathway in MM, including the cytoplasmic IκB data with 6 time-points at 0, 5, 10, 15, 20, and 30 minutes from western blot experiment in [11], [12], and the nuclear NFκB data with 6 time-points at 0, 10, 20, 30, 60, and 120 minutes from electrophoretic mobility shift assay (EMSA) in [11], [12] and flow cytometry experiment in our laboratory, which are shown in Figure S3. These dynamic time-course data are obtained by calculating the mean of all the corresponding data at each time-point. It is worth noting that the same time-points for the cytoplasmic IκB data and the nuclear NFκB data is not essential in the procedure of parameter estimation because the proposed optimization algorithm is able to handle this kind of data by minimizing the sum of square errors between the experimental data and the simulation data for all of the considered time points, as described in the section of parameter estimation in the previous text.

### ODEs system of NFκB pathway in MM

Here we describe the details for the ODEs system, but just list the equations for TNFα receptor sub-system as an example and the details for other three sub-systems are provided in Text S1 of Suppoting Information.

Module 1- TNFα receptor sub-system

This module describes the process from the binding of TNFα with its receptor to the formation of complex after recruitment.

TNFα: Equation (5) describes the changes on the concentration of TNFα due to binding (with rate 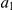) to its receptor TNFR1 and dissociation (with rate 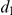) from the complex TNFα:TNFR1 (TNFR1C).
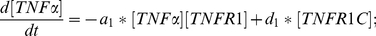
(5)


TNFR1: Equation (6) describes the changes on the concentration of TNFR1 due to binding (with rate 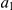) to its ligand TNFα and dissociation (with rate 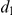) from the complex TNFR1C.
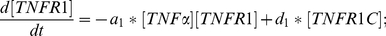
(6)


TNFR1C: Equation (7) describes the changes on the concentration of the complex TNFR1C due to association & dissociation mechanism between two teams of proteins, in which one is between TNFα and TNFR1 (with rates 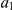 and 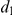) and another is between TNFR1C and TNFR1 adaptor (TNFR1A) (with rates 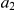 and 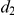).
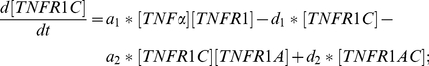
(7)


TNFR1A: Equation (8) describes the changes on the concentration of TNFR1A due to binding (with rate 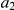) to the complex TNFR1C and dissociation (with rate 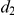) from the complex TNFR1:TNFR1A (TNFR1AC).
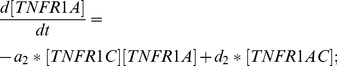
(8)


TNFR1AC: Equation (9) describes the changes on the concentration of the complex TNFR1AC due to association & dissociation mechanism between two teams of proteins, in which one is between TNFR1C and TNFR1A (with rates 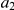 and 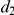) and another is between TNFR1AC and TRAFs (with rates 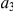 and 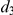). TRAFsC represents the complex TNFR1AC:TRAFs.
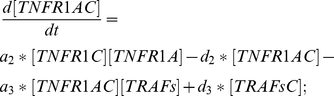
(9)


TRAFs: Equation (10) describes the changes on the concentration of TRAFs due to binding (with rate 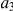) to the complex TNFR1AC and dissociation (with rate 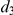) from the complex TRAFsC.
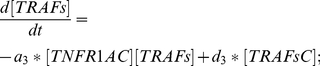
(10)


TRAFsC: Equation (11) describes the changes on the concentration of the complex TRAFsC due to association & dissociation mechanism between two teams of proteins, in which one is between TNFR1AC and TRAFs (with rates 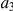 and 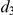) and another is between TRAFsC and IKKK (with rates 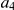 and 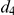). In addition, TRAFsC is also retrieved (with rate 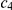) after catalysis from the complex TRAFsC:IKKK.
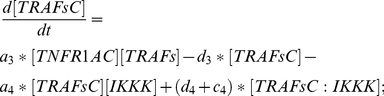
(11)


### Mechanisms of actions and drug modeling

Firstly, we introduce the mechanisms of actions for the considered drugs. In general, D1, D2 and D4 share the similar mechanism to inhibit the corresponding targets by binding mechanism. However, D3, with different mechanism, works to inhibit the degradation of IκBα by blocking the activity of proteasome. Based on these mechanisms, we got the drug modeling description as follows.

For D1, we assume it competitively inhibits TNFα with the same binding kinetics as that of the reaction involving TNFα and its receptor, that is, the binding rate is set as 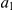 and the dissociation rate is set as 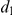. So, we add a new equation for D1 into the system, meanwhile we also modify an old equation for TNFa. For D2 and D4, it is similar with D1. The details of mechanism of actions and drug modeling for D1, D2 and D4 are provided in Text S2 of Supporting Information.

For D3 (i.e. BZM), it is the first therapeutic proteasome inhibitor to be tested in human and it has been approved in the US for treating relapsed MM. D3 works to inhibit the degradation of IκBα by blocking the activity of the proteasome. For simulating this drug's effect, we could not directly introduce an additional component to the system similarly as D1 because the degradation process of IκBα is not explicitly established in the ODEs model. By referring to [14], we can adjust the corresponding parameters in the terms for NFκB released after the degradation of IκBα, and the individual terms for IκBα and NFκB:IκBα molecules rescued from degradation. In order to describe the dose effect of D3 on the terms mentioned above, we introduce a Hill-type function to describe the inhibition rate for IκBα degradation by D3, which is defined as follows,
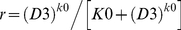
(12)Where 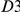 denotes the concentration of drug D3 and 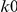 is set by 4 and 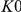 by 10e-10, and the corresponding curve can be seen from Figure S4 in which the corresponding concentration resulted in 50% inhibition is about 0.0055 µM. Referred to Figure 3, all of four terms related to the action of D3 are modified as follows, 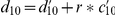, 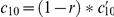, 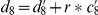 and 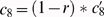, where 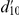, 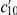, 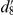 and 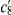 represent the parameters before modification, and 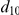, 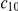, 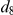 and 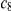 represent the parameters after modification.

## Supporting Information

Figure S1 Oscillation phenomenon. Oscillation phenomenon is presented in the model that constructed from literatures for cytoplasmic IKKp (A), cytoplasmic IκB (B) and nuclear NFκB (C). In the coordinate system, X and Y axes present time and concentration, respectively.
(0.27 MB TIF)

Figure S2 The parameters set obtained from the existed models can not fit the experimental data. Data fitting results for cytoplasmic IκB (A) and nuclear NFκB (B). Black box and solid curve represent the experimental data point and simulated results from the model with the collected parameters from literatures, respectively. In the coordinate system, X and Y axes present time and concentration, respectively.
(0.66 MB TIF)

Figure S3 Dynamic experimental data. The left sub-figure shows the western blot data for cytoplasmic IκB including five samples with up to six time-points, and the right sub-figure shows the EMSA data including two samples with three time-points and flow cytometry data with six time-points for nuclear NFκB. The above sub-figure shows the original experimental data and the corresponding quantified data based on the mean value is shown in the below sub-figure.
(0.52 MB TIF)

Figure S4 Inhibition rate curve for IκBα degradation by BZM. Based on the definition in Equation (12) of the main text under the assumption of Hill-type function, the presented curve can be used to describe the dose effect of BZM on the degradation of IκBα, in which the unit of BZM concentration in the X axes is µM. Note that the corresponding concentration resulted in 50% inhibition is about 0.0055 µM as pointed out in the curve.
(0.22 MB TIF)

Figure S5 Loewe synergy description based on classic IC50 - isobologram. According to the definition of the combination index in the right box, the drug combinations for point A, B and C indicate Loewe synergism, additive effect and antagonism, respectively; since the 50% isobologram from the left sub-figure is the red solid curve rather than the black dash-line or green dash-curve, it means that all of the combinations present Loewe synergy for drug 1 & drug 2.
(0.38 MB TIF)

Figure S6 Loewe isobolograms for different drug combinations in different cases of IC values. The blue contours in each sub-figure indicate the corresponding isobolograms, in which the column is for drug combination and the row is for inhibition percentage. For D2&D4 combination, in the case of IC75, a strong synergy effect can be found, however strong antagonism always can be seen in all of the cases of different IC values for D2&D3 combination. Note that this result is consistent with the result based on Bliss independence.
(0.39 MB TIF)

Table S1 Summary of the total 39 kinetic parameters in model.
(0.14 MB DOC)

Table S2 Summary of the initial concentrations in the model.
(0.07 MB DOC)

Text S1 The details of ODEs system for other three sub-systems in the model except for TNFÎ± receptor sub-system.
(0.30 MB DOC)

Text S2 The details of the mechanism of actions and the drug modeling for other three drugs in the model except for D3.
(0.08 MB DOC)
