# Supplementary figures and images for: Drug Inhibition Profile Prediction for NFκB Pathway in Multiple Myeloma
Source: PLoS One. 2011 Mar 7;6(3):e14750. doi: 10.1371/journal.pone.0014750 (PMC3051063; doi:10.1371/journal.pone.0014750)

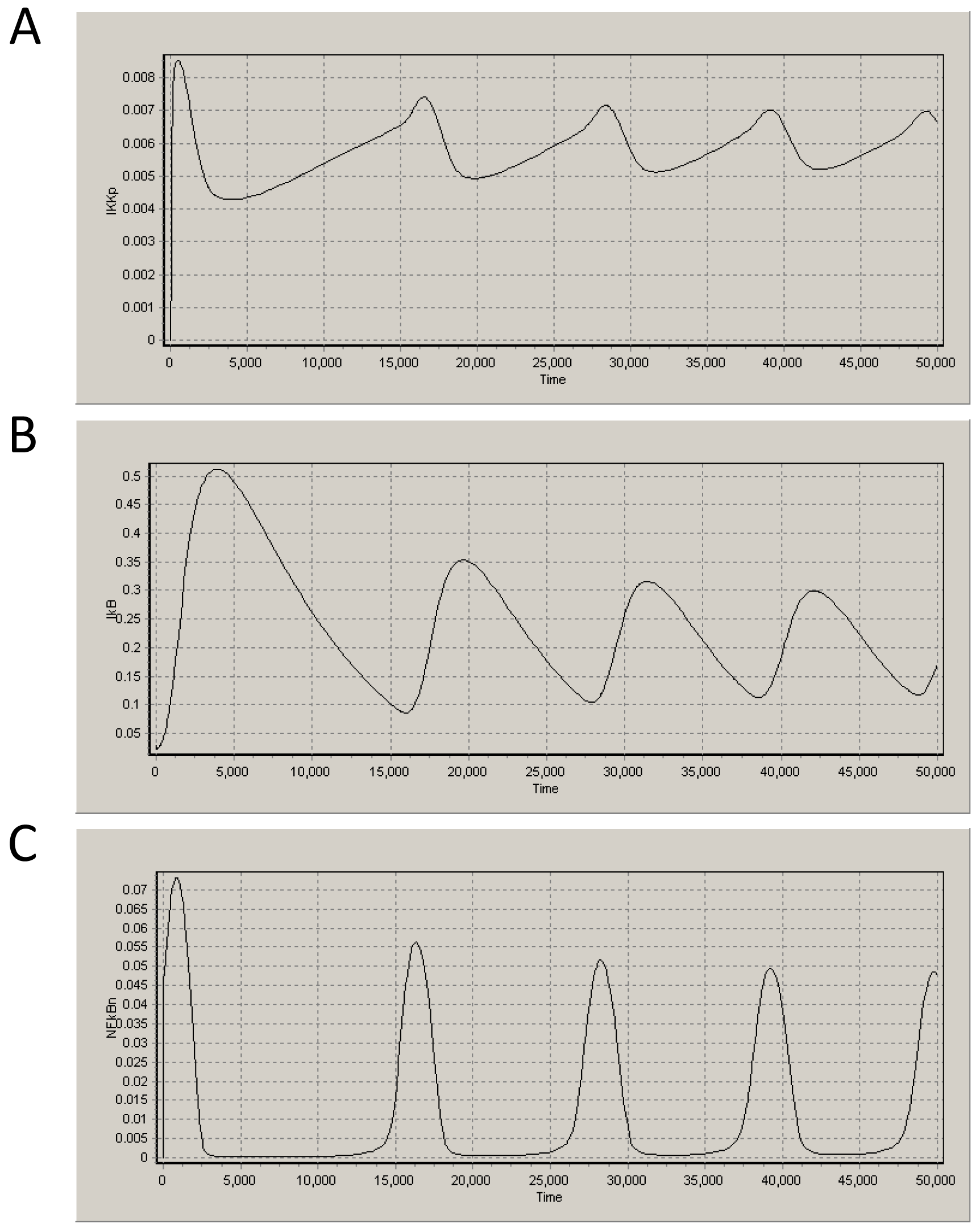

Supplement: Figure S1 — Oscillation phenomenon. Oscillation phenomenon is presented in the model that constructed from literatures for cytoplasmic IKKp (A), cytoplasmic IκB (B) and nuclear NFκB (C). In the coordinate system, X and Y axes present time and concentration, respectively. (0.27 MB TIF) [file pone.0014750.s001.tif]

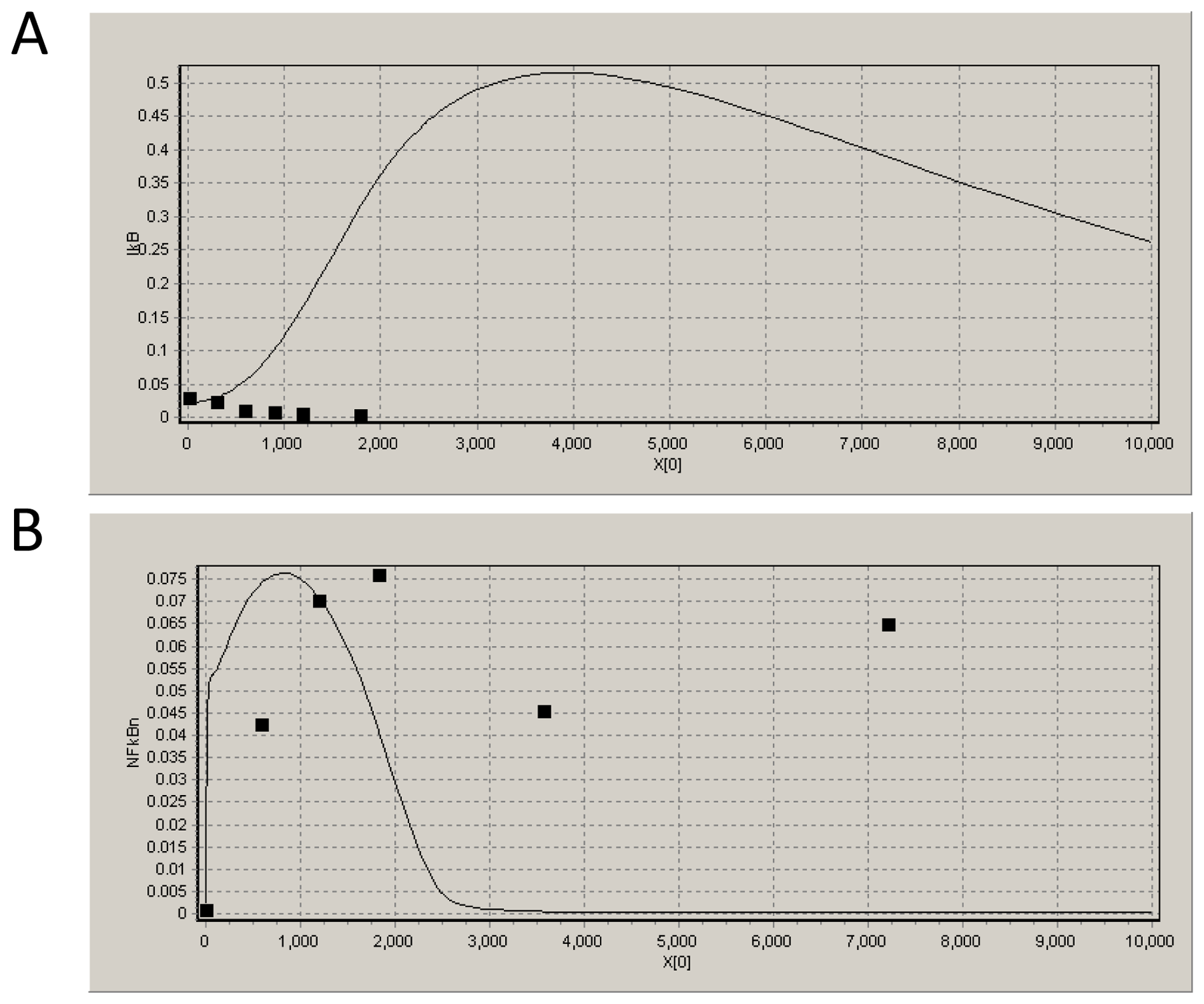

Supplement: Figure S2 — The parameters set obtained from the existed models can not fit the experimental data. Data fitting results for cytoplasmic IκB (A) and nuclear NFκB (B). Black box and solid curve represent the experimental data point and simulated results from the model with the collected parameters from literatures, respectively. In the coordinate system, X and Y axes present time and concentration, respectively. (0.66 MB TIF) [file pone.0014750.s002.tif]

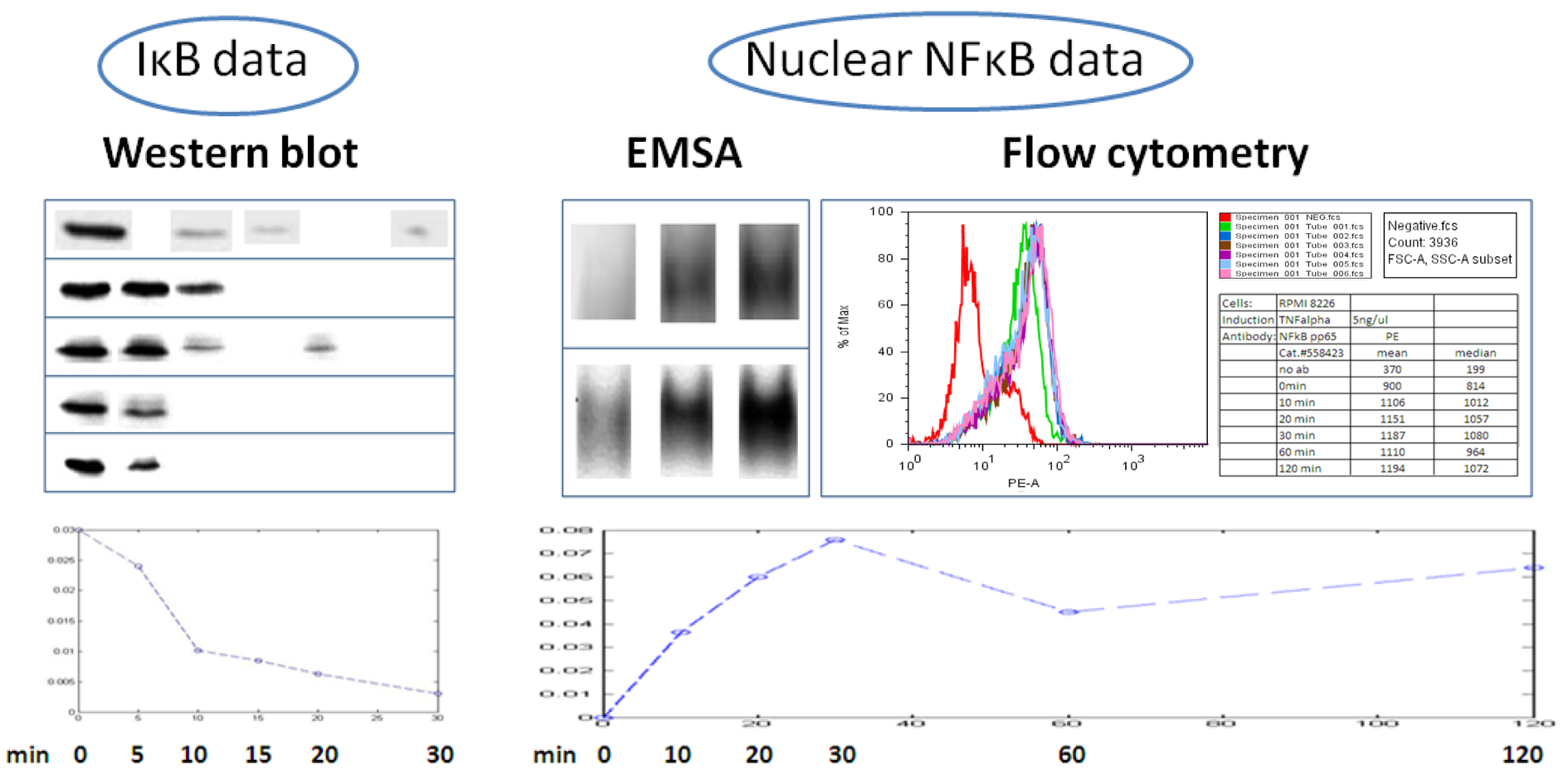

Supplement: Figure S3 — Dynamic experimental data. The left sub-figure shows the western blot data for cytoplasmic IκB including five samples with up to six time-points, and the right sub-figure shows the EMSA data including two samples with three time-points and flow cytometry data with six time-points for nuclear NFκB. The above sub-figure shows the original experimental data and the corresponding quantified data based on the mean value is shown in the below sub-figure. (0.52 MB TIF) [file pone.0014750.s003.tif]

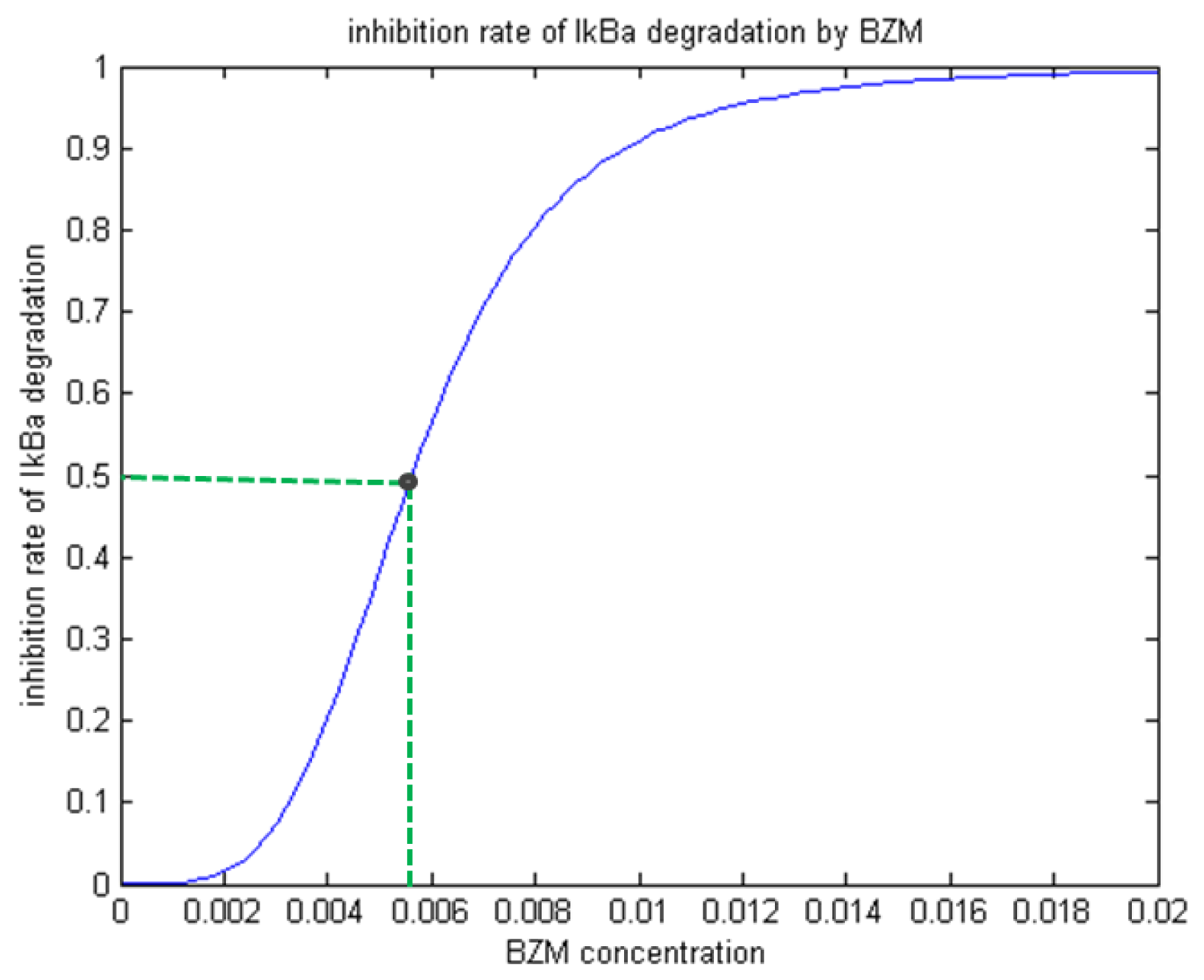

Supplement: Figure S4 — Inhibition rate curve for IκBα degradation by BZM. Based on the definition in Equation (12) of the main text under the assumption of Hill-type function, the presented curve can be used to describe the dose effect of BZM on the degradation of IκBα, in which the unit of BZM concentration in the X axes is µM. Note that the corresponding concentration resulted in 50% inhibition is about 0.0055 µM as pointed out in the curve. (0.22 MB TIF) [file pone.0014750.s004.tif]

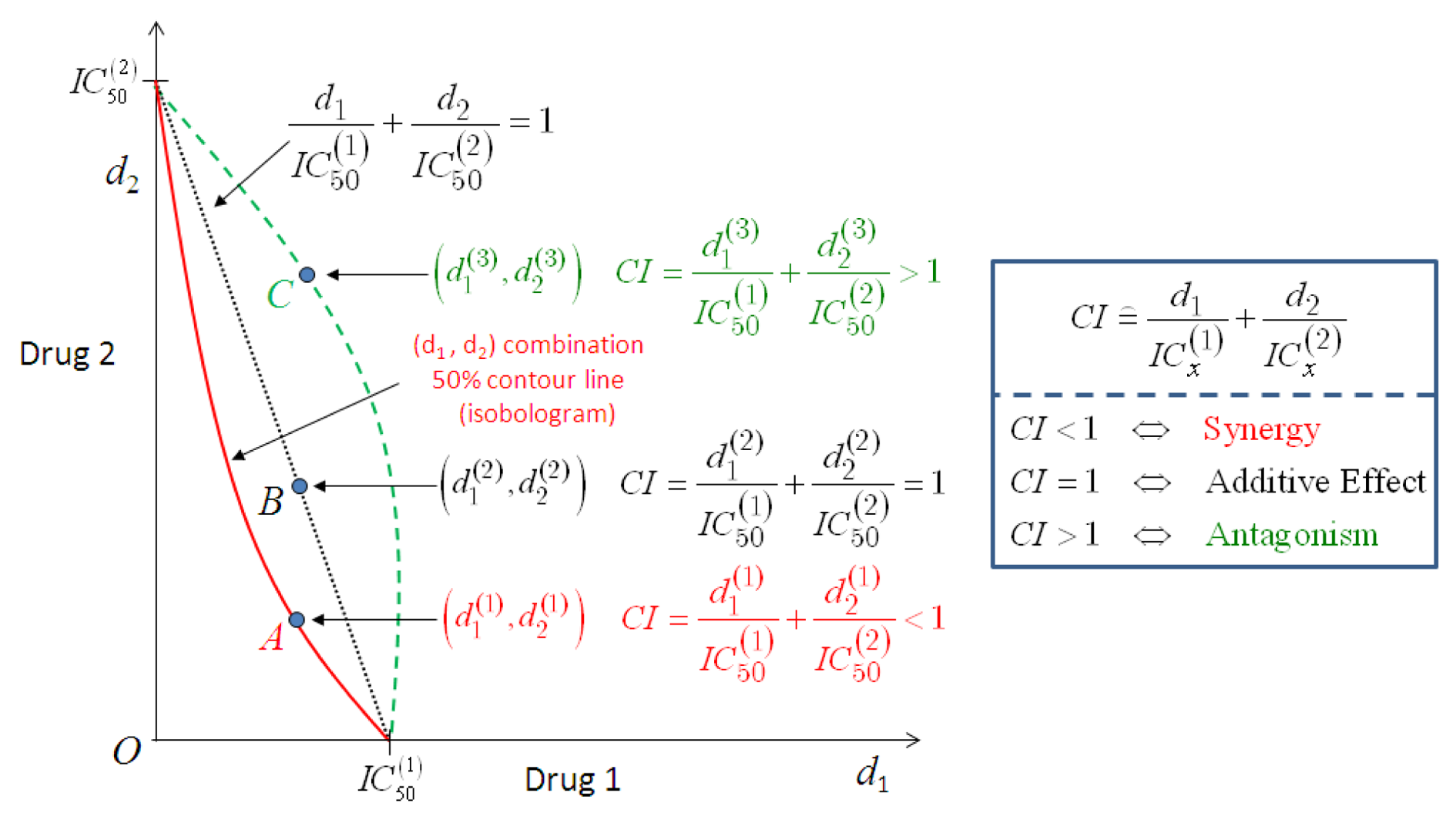

Supplement: Figure S5 — Loewe synergy description based on classic IC50 - isobologram. According to the definition of the combination index in the right box, the drug combinations for point A, B and C indicate Loewe synergism, additive effect and antagonism, respectively; since the 50% isobologram from the left sub-figure is the red solid curve rather than the black dash-line or green dash-curve, it means that all of the combinations present Loewe synergy for drug 1 & drug 2. (0.38 MB TIF) [file pone.0014750.s005.tif]

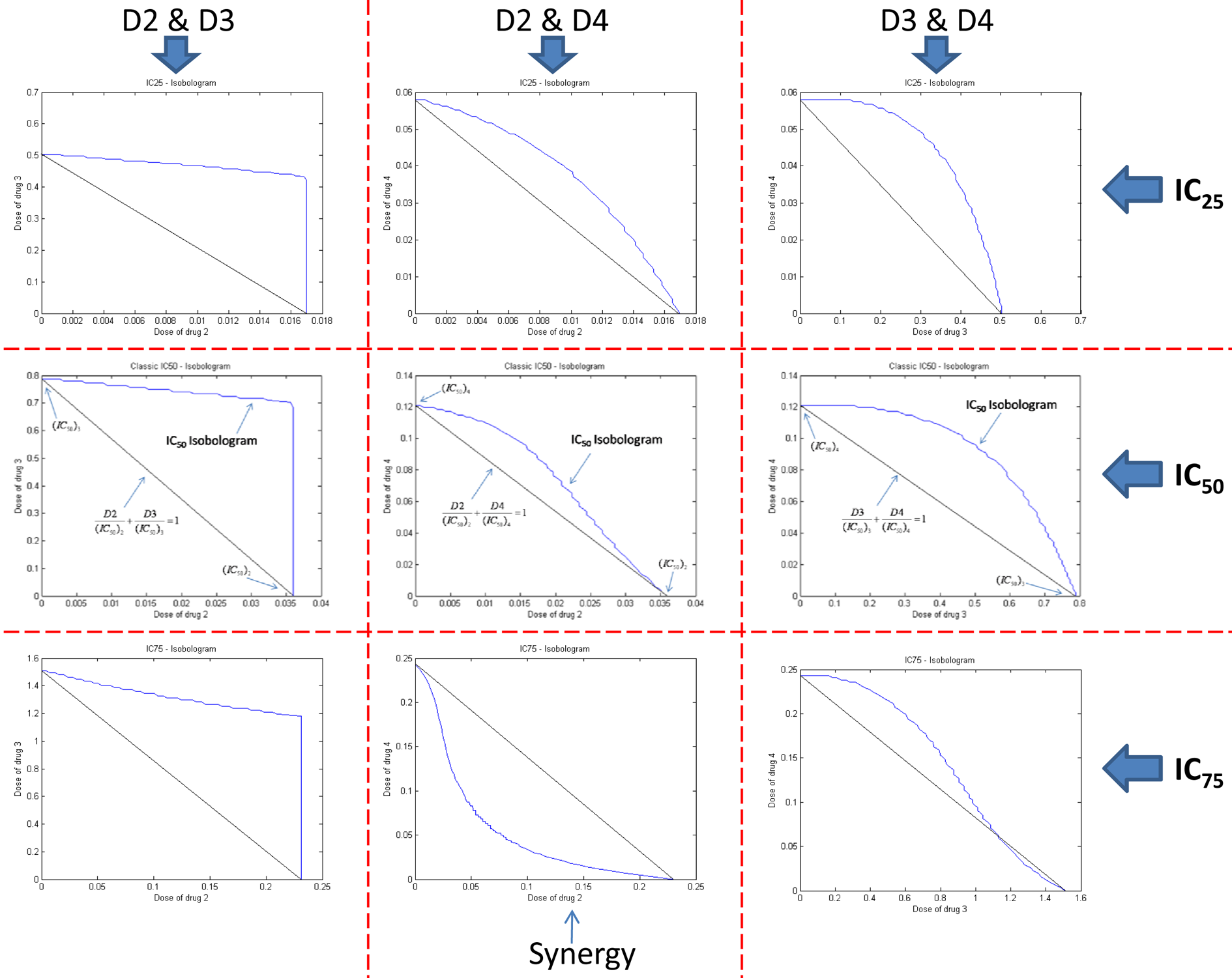

Supplement: Figure S6 — Loewe isobolograms for different drug combinations in different cases of IC values. The blue contours in each sub-figure indicate the corresponding isobolograms, in which the column is for drug combination and the row is for inhibition percentage. For D2&D4 combination, in the case of IC75, a strong synergy effect can be found, however strong antagonism always can be seen in all of the cases of different IC values for D2&D3 combination. Note that this result is consistent with the result based on Bliss independence. (0.39 MB TIF) [file pone.0014750.s006.tif]
