# Supplementary material for: Drug Inhibition Profile Prediction for NFκB Pathway in Multiple Myeloma
Source: PLoS One. 2011 Mar 7;6(3):e14750. doi: 10.1371/journal.pone.0014750 (PMC3051063; doi:10.1371/journal.pone.0014750)
Supplement: Table S1 — Summary of the total 39 kinetic parameters in model. (0.14 MB DOC) [file pone.0014750.s007.doc]

**Table S1. Summary of the model parameters.**

Table 1. Summary of the total 39 kinetic parameters in model.

| **Description** | **Symbol** | **Value** | **Unit** | **References** |
| --- | --- | --- | --- | --- |
| **Module 1 – TNF**α **receptor sub-system** | | | | |
| TNFα:TNFR1 association | a1 | 18.3331 | $\text{μM}^{-1}\text{s}^{-1}$ | [1] |
| TNFα:TNFR1 dissociation | d1 | 0.002 | $\text{s}^{-1}$ | [1] |
| TNFR1C:TNFR1A association | a2 | 0.185 | $\text{μM}^{-1}\text{s}^{-1}$ | [1] |
| TNFR1C:TNFR1A dissociation | d2 | 0.00125 | $\text{s}^{-1}$ | [1] |
| TNFR1AC:TRAFs association | a3 | 0.185 | $\text{μM}^{-1}\text{s}^{-1}$ | [1] |
| TNFR1AC:TRAFs dissociation | d3 | 0.00125 | $\text{s}^{-1}$ | [1] |
| **Module 2 – IKK phosphorylation cascade sub-system** | | | | |
| TRAFsC:IKKK association | a4 | 10 | $\text{s}^{-1}$ | [1] |
| TRAFsC:IKKK dissociation | d4 | 0.5 | $\text{s}^{-1}$ | [1] |
| TRAFsC:IKKK catalysis | c4 | 0.1 | $\text{μM}^{-1}\text{s}^{-1}$ | [1] |
| IKKKp:Phosphatase1 association | a5 | 10 | $\text{s}^{-1}$ | [1] |
| IKKKp:Phosphatase1 dissociation | d5 | 0.5 | $\text{s}^{-1}$ | [1] |
| IKKKp:Phosphatase1 catalysis | c5 | 0.1 | $\text{μM}^{-1}\text{s}^{-1}$ | [1] |
| IKKKp:IKK association | a6 | 10 | $\text{s}^{-1}$ | [1] |
| IKKKp:IKK dissociation | d6 | 0.5 | $\text{s}^{-1}$ | [1] |
| IKKKp:IKK catalysis | c6 | 0.1 | $\text{μM}^{-1}\text{s}^{-1}$ | [1] |
| IKKp:Phosphatase2 association | a7 | 10 | $\text{s}^{-1}$ | [1] |
| IKKp:Phosphatase2 dissociation | d7 | 0.5 | $\text{s}^{-1}$ | [1] |
| IKKp:Phosphatase2 catalysis | c7 | 0.1 | $\text{s}^{-1}$ | [1] |
| **Module 3 – cytoplasmic IKK-IκB-NFκB sub-system** | | | | |
| IKKp:IκB:NFκB association | a8 | 5.507750e-1 | $\text{μM}^{-1}\text{s}^{-1}$ | Estimated |
| IKKp:IκB:NFκB dissociation | d8 | 7.812500e-5 | $\text{s}^{-1}$ | Estimated |
| IKKp:IκB:NFκB catalysis | c8 | 2.805000e-3 | $\text{s}^{-1}$ | Estimated |
| IκB:NFκB association | a9 | 9.875000e-2 | $\text{μM}^{-1}\text{s}^{-1}$ | Estimated |
| IκB:NFκB dissociation | d9 | 3.125000e-4 | $\text{s}^{-1}$ | Estimated |
| IKKp:IκB association | a10 | 4.488750e-1 | $\text{μM}^{-1}\text{s}^{-1}$ | Estimated |
| IKKp:IκB dissociation | d10 | 7.812500e-4 | $\text{s}^{-1}$ | Estimated |
| IKKp:IκB catalysis | c10 | 1.017500e-4 | $\text{s}^{-1}$ | Estimated |
| IκB degradation | dg1 | 1.412500e-4 | $\text{s}^{-1}$ | Estimated |
| IκB:NFκB degradation | dg2 | 1.125000e-5 | $\text{s}^{-1}$ | Estimated |
| **Module 4 – nuclear IκB-NFκB sub-system** | | | | |
| IκBn:NFκBn association | a11 | 9.000000e-2 | $\text{μM}^{-1}\text{s}^{-1}$ | Estimated |
| IκBn:NFκBn dissociation | d11 | 3.525000e-4 | $\text{s}^{-1}$ | Estimated |
| NFκB nuclear import | i1 | 2.812500e-3 | $\text{s}^{-1}$ | Estimated |
| NFκB nuclear export | e1 | 1.500000e-4 | $\text{s}^{-1}$ | Estimated |
| IκB nuclear import | i2 | 1.556250e-3 | $\text{s}^{-1}$ | Estimated |
| IκB nuclear export | e2 | 8.300000e-4 | $\text{s}^{-1}$ | Estimated |
| IκB:NFκB nuclear export | e3 | 1.982887e-2 | $\text{s}^{-1}$ | Estimated |
| IκB mRNA degradation | dg3 | 3.675000e-4 | $\text{s}^{-1}$ | Estimated |
| IκB constitutive mRNA synthesis | tr1 | 1.001000e-6 | $\text{μMs}^{-1}$ | Estimated |
| IκB inducible mRNA synthesis | tr2 | 3.093750e-3 | $\text{μM}^{-1}\text{s}^{-1}$ | Estimated |
| IκB translation rate | tr3 | 9.096500e-4 | $\text{s}^{-1}$ | Estimated |

**Reference:**

1. Park SG, Lee T, Cho K-H, Kang HY, Park. K, et al. (2006) The influence of the signal dynamics of activated form of IKK on NF-kB and anti-apoptotic expressions: A systems biology approach. FEBS Letters 580: 822-830.
