# Supplementary material for: Drug Inhibition Profile Prediction for NFκB Pathway in Multiple Myeloma
Source: PLoS One. 2011 Mar 7;6(3):e14750. doi: 10.1371/journal.pone.0014750 (PMC3051063; doi:10.1371/journal.pone.0014750)
Supplement: Table S2 — Summary of the initial concentrations in the model. (0.07 MB DOC) [file pone.0014750.s008.doc]

**Table S2. Summary of the initial concentrations in the model.**

Table 2. Summary of the total 11 initial concentrations in the model.

| **Molecule** | **Value** | **Unit** | **References** |
| --- | --- | --- | --- |
| TNFα | 0.2 | $\text{μM}$ | [1] |
| TNFR1 | 0.16667 | $\text{μM}$ | [1] |
| TNFR1A (TNFR1 Adaptor) | 0.1 | $\text{μM}$ | [1] |
| TRAFs | 0.33389 | $\text{μM}$ | [1] |
| IKKK | 0.04522 | $\text{μM}$ | [1] |
| Phosphatase1 | 0.0835 | $\text{μM}$ | [1] |
| IKK | 0.02278 | $\text{μM}$ | [1] |
| Phosphatase2 | 0.0113 | $\text{μM}$ | [1] |
| IκB:NFκB | 0.04 | $\text{μM}$ | [2, 3] |
| NFκB | 0.06 | $\text{μM}$ | [2, 3] |
| IκB | 0.03 | $\text{μM}$ | [2, 3] |

**References:**

1. Park SG, Lee T, Cho K-H, Kang HY, Park. K, et al. (2006) The influence of the signal dynamics of activated form of IKK on NF-kB and anti-apoptotic expressions: A systems biology approach. FEBS Letters 580: 822-830.

2. Hoffmann A, Levchenko A, Scott ML, Baltimore D (2002) The IkappaB-NF-kappaB signaling module: temporal control and selective gene activation. Science 298: 1241-1245.

3. Sung MH, Simon R (2004) In silico simulation of inhibitor drug effects on nuclear factor-kappaB pathway dynamics. Mol Pharmacol 66: 70-75.
