# Supplementary material for: Drug Inhibition Profile Prediction for NFκB Pathway in Multiple Myeloma
Source: PLoS One. 2011 Mar 7;6(3):e14750. doi: 10.1371/journal.pone.0014750 (PMC3051063; doi:10.1371/journal.pone.0014750)
Supplement: Text S1 — The details of ODEs system for other three sub-systems in the model except for TNFÎ± receptor sub-system. (0.30 MB DOC) [file pone.0014750.s009.doc]

**Text S1. The details of ODEs system for other three sub-systems in the model except for TNFα receptor sub-system.**

Module 2- IKK phosphorylation cascade sub-system

This module describes the phosphorylation cascade process from IKKK to IKK.

IKKK: Equation (1) describes the changes on the concentration of IKKK due to binding (with rate $a_{4}$) to the complex TRAFsC and dissociation (with rate $d_{4}$) from the complex TRAFsC:IKKK. In addition, IKKK is also retrieved (with rate $c_{5}$) from the complex IKKKp:Phosphatase1 after catalysis.

$\frac{d[IKKK]}{dt}=-a_{4}*[TRAFsC][IKKK]+d_{4}*[TRAFsC:IKKK]+c_{5}*[IKKKp:Phosphatase1];$ (1)

TRAFsC:IKKK: Equation (2) describes the changes on the concentration of TRAFs due to association & dissociation mechanism between TRAFsC and IKKK (with rates $a_{4}$ and $d_{4}$).

$\frac{d[TRAFsC:IKKK]}{dt}=a_{4}*[TRAFsC][IKKK]-d_{4}*[TRAFsC:IKKK];$ (2)

IKKKp: Equation (3) describes the changes on the concentration of IKKKp due to association & dissociation mechanism between two teams of proteins, in which one is between IKKKp and Phosphatase1 (with rates $a_{5}$ and $d_{5}$) and another is between IKKKp and IKK (with rates $a_{6}$ and $d_{6}$). In addition, IKKKp is phosphorylated (with rate $c_{4}$) from IKKK after catalysis of the complex TRAFsC:IKKK, and is also retrieved (with rate $c_{6}$) from the complex IKKKp:IKK after catalysis.

$\begin{matrix} \frac{d[IKKKp]}{dt}=c_{4}*[TRAFsC:IKKK]-a_{5}*[IKKKp][Phosphatase1]+d_{5}*[IKKKp:Phosphatase1] \\ -a_{6}*[IKKKp][IKK]+(d_{6}+c_{6})*[IKKKp:IKK]; \end{matrix}$ (3)

IKKKp:Phosphatase1: Equation (4) describes the changes on the concentration of the complex IKKKp:Phosphatase1 due to association & dissociation mechanism between IKKKp and Phosphatase1 (with rates $a_{5}$ and $d_{5}$).

$\frac{d[IKKKp:Phosphatase1]}{dt}=a_{5}*[IKKKp][Phosphatase1]-d_{5}*[IKKKp:Phosphatase1];$ (4)

Phosphatase1: Equation (5) describes the changes on the concentration of Phosphatase1 due to association & dissociation mechanism between IKKKp and Phosphatase1 (with rates $a_{5}$ and $d_{5}$). In addition, Phosphatase1 is also retrieved (with rate $c_{5}$) from the complex IKKKp:Phosphatase1 after catalysis.

$\frac{d[Phosphatase1]}{dt}=-a_{5}*[IKKKp][Phosphatase1]+(d_{5}+c_{5})*[IKKKp:Phosphatase1];$ (5)

IKK: Equation (6) describes the changes on the concentration of IKK due to association & dissociation mechanism between IKKKp and IKK (with rates $a_{6}$ and $d_{6}$). In addition, IKK is also dephosphorylated (with rate $c_{7}$) from IKKp after catalysis of the complex IKKp:Phosphatase2.

$\frac{d[IKK]}{dt}=-a_{6}*[IKKKp][IKK]+d_{6}*[IKKKp:IKK]+c_{7}*[IKKp:Phosphatase2];$ (6)

IKKKp:IKK: Equation (7) describes the changes on the concentration of the complex IKKKp:IKK due to association & dissociation mechanism between IKKKp and IKK (with rates $a_{6}$ and $d_{6}$). In addition, it is also decompounded (with rate $c_{6}$) after catalysis.

$\frac{d[IKKKp:IKK]}{dt}=a_{6}*[IKKKp][IKK]-(d_{6}+c_{6})*[IKKKp:IKK];$ (7)

IKKp:Phosphatase2: Equation (8) describes the changes on concentration of the complex IKKp:Phosphatase2 due to association & dissociation mechanism between IKKp and Phosphatase2 (with rates $a_{7}$ and $d_{7}$). In addition, it is also decompounded (with rate $c_{7}$) after catalysis.

$\frac{d[IKKp:Phosphatase2]}{dt}=a_{7}*[IKKp][Phosphatase2]-(d_{7}+c_{7})*[IKKp:Phosphatase2];$ (8)

Phosphatase2: Equation (9) describes the changes on the concentration of Phosphatase2 due to binding (with rate $a_{7}$) to the IKKp and dissociation (with rate $d_{7}$) from the complex IKKp:Phosphatase2. In addition, it is also retrieved (with rate $c_{7}$) from the complex IKKp:Phosphatase2 after catalysis.

$\frac{d[Phosphatase2]}{dt}=-a_{7}*[IKKp][Phosphatase2]+(d_{7}+c_{7})*[IKKp:Phosphatase2];$ (9)

IKKp: Equation (10) describes the changes on the concentration of IKKp due to association & dissociation mechanism between three teams of proteins, in which one is between IKKp and Phosphatase2 (with rates $a_{7}$ and $d_{7}$), another is between IKKp and the complex IκB:NFκB (with rates $a_{8}$ and $d_{8}$), and the other is between IKKp and IκB (with rates $a_{10}$ and $d_{10}$). In addition, IKKp is phosphorylated (with rate $c_{6}$) from IKK after catalysis of the complex IKKKp:IKK, and is also retrieved (with rates $c_{8}$ and $c_{10}$) from the complex IKKp:IκB: NFκB and IKKp:IκB after catalysis, respectively.

$\begin{matrix} \frac{d[IKKp]}{dt}=c_{6}*[IKKKp:IKK]-a_{7}*[IKKp][Phosphatase2]+d_{7}*[IKKp:Phosphatase2] \\ -a_{8}*[IKKp][I\kappa B:NF\kappa B]+(d_{8}+c_{8})*[IKKp:I\kappa B:NF\kappa B] \\ -a_{10}*[IKKp][I\kappa B]+(d_{10}+c_{10})*[IKKp:I\kappa B]; \end{matrix}$ (10)

Modules 3 – cytoplasmic IKK-IκB-NFκB sub-system

This module describes the process of NFκB releasing from its inhibitor IκBα after the degradation of IκBα by phosphorylated IKK in cytoplasm.

IκB:NFκB: Equation (11) describes the changes on the concentration of the complex IκB:NFκB due to association & dissociation mechanism between two teams of proteins, in which one is between IκB:NFκB and IKKp (with rates $a_{8}$ and $d_{8}$), and another is between IκB and NFκB (with rates $a_{9}$ and $d_{9}$). In addition, it is retrieved by translocation of the nuclear complex IκBn:NFκBn from nucleus to cytoplasm (with rate $e_{3}$), meanwhile it is also degraded (with rates $dg_{2}$).

$\begin{matrix} \frac{d[I\kappa B:NF\kappa B]}{dt}=-a_{8}*[IKKp][I\kappa B:NF\kappa B]+d_{8}*[IKKp:I\kappa B:NF\kappa B]+a_{9}*[I\kappa B][NF\kappa B] \\ -d_{9}*[I\kappa B:NF\kappa B]+e_{3}*[I\kappa Bn:NF\kappa Bn]-dg_{2}*[I\kappa B:NF\kappa B]; \end{matrix}$ (11)

IKKp:IκB:NFκB: Equation (12) describes the changes on the concentration of the complex IKKp:IκB:NFκB due to association & dissociation mechanism between IKKp and IκB:NFκB (with rates $a_{8}$ and $d_{8}$). In addition, it is also decompounded (with rate $c_{8}$) after catalysis.

$\frac{d[IKKp:I\kappa B:NF\kappa B]}{dt}=a_{8}*[IKKp][I\kappa B:NF\kappa B]-(d_{8}+c_{8})*[IKKp:I\kappa B:NF\kappa B];$ (12)

NFκB: Equation (13) describes the changes on the concentration of cytoplasmic NFκB due to association & dissociation mechanism between IκB and NFκB (with rates $a_{9}$ and $d_{9}$). In addition, it is retrieved from the complex IKKp:IκB:NFκB after catalysis (with rate $c_{8}$) and the complex IκB:NFκB after IκB degradation (with rate $dg_{2}$), and is also retrieved by translocation of nuclear NFκB (NFκBn) from nucleus to cytoplasm (with rate $e_{1}$), meanwhile it is decreased by translocation from cytoplasm to nucleus (with rates $i_{1}$).

$\begin{matrix} \frac{d[NF\kappa B]}{dt}=c_{8}*[IKKp:I\kappa B:NF\kappa B]-a_{9}*[I\kappa B][NF\kappa B]+d_{9}*[I\kappa B:NF\kappa B] \\ +dg_{2}*[I\kappa B:NF\kappa B]-i_{1}*[NF\kappa B]+e_{1}*[NF\kappa Bn]; \end{matrix}$ (13)

IκB: Equation (14) describes the changes on the concentration of the cytoplasmic free IκB due to association & dissociation mechanism between two teams of proteins, in which one is between IκB and NFκB (with rates $a_{9}$ and $d_{9}$), and another is between IκB and IKKp (with rates $a_{10}$ and $d_{10}$). In addition, it is retrieved by translocation of nuclear IκB (IκBn) from nucleus to cytoplasm (with rate $e_{2}$), but decreased by translocation from cytoplasm to nucleus (with rates $i_{2}$). Moreover, it is also generated from transcription (with rate $tr_{3}$) , meanwhile degraded (with rate $dg_{1}$).

$\begin{matrix} \frac{d[I\kappa B]}{dt}=-a_{9}*[I\kappa B][NF\kappa B]+d_{9}*[I\kappa B:NF\kappa B]-a_{10}*[IKKp][I\kappa B]+d_{10}*[IKKp:I\kappa B] \\ -i_{2}*[I\kappa B]+e_{2}*[I\kappa Bn]-dg_{1}*[I\kappa B]+tr_{3}*[I\kappa Bt]; \end{matrix}$ (14)

IKKp:IκB: Equation (15) describes the changes on concentration of the complex IKKp:IκB due to association & dissociation mechanism between IKKp and IκB (with rates $a_{10}$ and $d_{10}$). In addition, it is also decompounded (with rate $c_{10}$) after catalysis.

$\frac{d[IKKp:I\kappa B]}{dt}=a_{10}*[IKKp][I\kappa B]-(d_{10}+c_{10})*[IKKp:I\kappa B];$ (15)

Module 4 – nuclear IκB-NFκB sub-system

This module describes the process of nuclear NFκB production involving the translocation of NFκB from cytoplasm to nucleus and a negative feedback loop on the transcription of IκB by nuclear NFκB.

IκBn:NFκBn: Equation (16) describes the changes on the concentration of the nuclear complex IκB:NFκB (IκBn:NFκBn) due to association & dissociation mechanism between nuclear IκB (IκBn) and nuclear NFκB (NFκBn) (with rates $a_{11}$ and $d_{11}$). In addition, it is also decreased by the translocation from nucleus to cytoplasm (with rate $e_{3}$).

$\frac{d[I\kappa Bn:NF\kappa Bn]}{dt}=a_{11}*[I\kappa Bn][NF\kappa Bn]-d_{11}*[I\kappa Bn:NF\kappa Bn]-e_{3}*[I\kappa Bn:NF\kappa Bn];$ (16)

IκBn: Equation (17) describes the changes on the concentration of the nuclear IκB (IκBn) due to binding (with rate $a_{11}$) to nuclear NFκB (NFκBn) and dissociation (with rate $d_{11}$) from the nuclear complex IκB:NFκB (IκBn:NFκBn). In addition, it is increased by the translocation of IκB from cytoplasm to nucleus (with rate $i_{2}$), and also decreased by the translocation of itself from nucleus to cytoplasm (with rate $e_{2}$).

$\frac{d[I\kappa Bn]}{dt}=-a_{11}*[I\kappa Bn][NF\kappa Bn]+d_{11}*[I\kappa Bn:NF\kappa Bn]+i_{2}*[I\kappa B]-e_{2}*[I\kappa Bn];$ (17)

NFκBn: Equation (18) describes the changes on the concentration of the nuclear NFκB (NFκBn) due to binding (with rate $a_{11}$) to nuclear IκB (IκBn) and dissociation (with rate $d_{11}$) from the nuclear complex IκB:NFκB (IκBn:NFκBn). In addition, it is increased with the translocation of NFκB from cytoplasm to nucleus (with rate $i_{1}$), but decreased with the translocation of itself from nucleus to cytoplasm (with rate $e_{1}$).

$\frac{d[NF\kappa Bn]}{dt}=-a_{11}*[I\kappa Bn][NF\kappa Bn]+d_{11}*[I\kappa Bn:NF\kappa Bn]+i_{1}*[NF\kappa B]-e_{1}*[NF\kappa Bn];$ (18)

IκBt: Equation (19) describes the changes on the concentration of IκB mRNA due to constitutive mRNA transcription (with rate $tr_{1}$), inducible mRNA transcription by nuclear NFκB (NFκBn) (with rate $tr_{2}$), and itself degradation (with rate $dg_{3}$).

$\frac{d[I\kappa Bt]}{dt}=tr_{1}+tr_{2}*[NF\kappa Bn]*[NF\kappa Bn]-dg_{3}*[I\kappa Bt];$ (19)
