# Supplementary material for: Drug Inhibition Profile Prediction for NFκB Pathway in Multiple Myeloma
Source: PLoS One. 2011 Mar 7;6(3):e14750. doi: 10.1371/journal.pone.0014750 (PMC3051063; doi:10.1371/journal.pone.0014750)
Supplement: Text S2 — The details of the mechanism of actions and the drug modeling for other three drugs in the model except for D3. (0.08 MB DOC) [file pone.0014750.s010.doc]

**Text S2. The details of the mechanism of actions and the drug modeling for other three drugs in the model except for D3.**

D1 (i.e. Infliximab) is a monoclonal antibody against TNFα and it has been approved by the FDA for the treatment of many diseases. It works by binding to TNFα, so it can inhibit the NFκB pathway. In this study, we assume that D1 competitively inhibit TNFα with binding kinetics which is the same as that of the natural reaction involving TNFα and TNFR1, that is, the binding rate is set as $a_{1}$ and the dissociation rate is set as $d_{1}$, as seen from Figure 3 in the main text. So, we can add two new equations for drug D1 and the complex TNFα:D1 into the ODEs model, meanwhile we also need to modify an old equation for TNFα. These three equations are listed as follows.

$\frac{d[D1]}{dt}=-a_{1}*[D1][TNF\alpha]+d_{1}*[TNF\alpha:D1];$ (1) $\frac{d[TNF\alpha:D1]}{dt}=a_{1}*[D1][TNF\alpha]-d_{1}*[TNF\alpha:D1];$ (2)

$\frac{d[TNF\alpha]}{dt}=-a_{1}*[TNF\alpha][TNFR1]+d_{1}*[TNFR1C]-a_{1}*[D1][TNF\alpha]+d_{1}*[TNF\alpha:D1].$ (3)

For D2 (i.e. ATO), it is an inorganic compound and a traditional Chinese medicine, and it also has been approved by the FDA for the treatment of certain leukemias. Based on the information from DrugBank website, ATO inhibits the NFκB pathway by targeting the protein IKKp (phosphorylated IKK). So, the effect of administering D2 is modeled in a similar manner as D1, i.e. D2 is assumed to competitively inhibit IKKp with the same binding kinetics as the reaction between IKKp and the cytoplasmic complex IκB:NFκB. Based on the parameter symbol description in Figure 2 of the main text, the binding and dissociation rate between D2 and IKKp are set as $a_{8}$ and $d_{8}$, respectively. So, we can add two new equations for drug D2 and the complex IKKp:D2 into the ODEs model, meanwhile we also need to modify an old equation for IKKp. These three equations are listed as follows.

$\frac{d[D2]}{dt}=-a_{8}*[IKKp][D2]+d_{8}*[IKKp:D2];$ (4)

$\frac{d[IKKp:D2]}{dt}=a_{8}*[IKKp][D2]-d_{8}*[IKKp:D2];$ (5)

$\begin{matrix} \frac{d[IKKp]}{dt}=c_{6}*[IKKKp:IKK]-a_{7}*[IKKp][Phosphatase2]+d_{7}*[IKKp:Phosphatase2] \\ -a_{8}*[IKKp][I\kappa B:NF\kappa B]+(d_{8}+c_{8})*[IKKp:I\kappa B:NF\kappa B] \\ -a_{10}*[IKKp][I\kappa B]+(d_{10}+c_{10})*[IKKp:I\kappa B] \\ -a_{8}*[IKKp][D2]+d_{8}*[IKKp:D2]; \end{matrix}$ (6)

For D4 (i.e. A238L), an IκB-like protein which can act as a homologue of IκBα, it inhibits the activity of NFκB by directly binding to cytoplasmic NFκB to block its translocation from cytoplasm to nucleus. So we model the effect of D4 by binding mechanism similar as previously, i.e. it competitively inhibits the cytoplasmic NFκB with the same binding kinetics as the reaction between NFκB and IκBα with rate constants $a_{9}$ and $d_{9}$. Therefore, we can add two new equations for drug D4 and the complex NFκB:D4 into the ODEs model, meanwhile we also need to modify an old equation for the cytoplasmic NFκB. These three equations are listed as follows.

$\frac{d[D4]}{dt}=-a_{9}*[NF\kappa B][D4]+d_{8}*[NF\kappa B:D4];$ (7)

$\frac{d[NF\kappa B:D4]}{dt}=a_{9}*[NF\kappa B][D4]-d_{8}*[NF\kappa B:D4];$ (8)

$\begin{matrix} \frac{d[NF\kappa B]}{dt}=c_{8}*[IKKp:I\kappa B:NF\kappa B]-a_{9}*[I\kappa B][NF\kappa B]+d_{9}*[I\kappa B:NF\kappa B] \\ +dg_{2}*[I\kappa B:NF\kappa B]-i_{1}*[NF\kappa B]+e_{1}*[NF\kappa Bn] \\ -a_{9}*[NF\kappa B][D4]+d_{9}*[NF\kappa B:D4]. \end{matrix}$ (9)
